# Tumor microenvironment remodeling after neoadjuvant immunotherapy in non-small cell lung cancer revealed by single-cell RNA sequencing

**DOI:** 10.1186/s13073-023-01164-9

**Published:** 2023-03-03

**Authors:** Junjie Hu, Lele Zhang, Haoran Xia, Yilv Yan, Xinsheng Zhu, Fenghuan Sun, Liangdong Sun, Shuangyi Li, Dianke Li, Jin Wang, Ya Han, Jing Zhang, Dongliang Bian, Huansha Yu, Yan Chen, Pengyu Fan, Qiang Ma, Gening Jiang, Chenfei Wang, Peng Zhang

**Affiliations:** 1 Department of Thoracic Surgery, Shanghai Pulmonary Hospital, Tongji University School of Medicine, No. 507 Zhengmin Road, Shanghai, 200433 China; 2 Central Laboratory, Shanghai Pulmonary Hospital, Tongji University School of Medicine, Shanghai, 200433 China; 3 Key Laboratory of Spine and Spinal Cord Injury Repair and Regeneration of Ministry of Education, Department of Orthopedics, Tongji Hospital, School of Life Science and Technology, Tongji University, 1239 Siping Road, Shanghai, 200092 China; 4 Experimental Animal Center, Shanghai Pulmonary Hospital, Tongji University School of Medicine, Shanghai, 200433 China; 5 Frontier Science Center for Stem Cells, School of Life Science and Technology, Tongji University, Shanghai, 200092 China; 6 The 1st School of Medicine, The 1st Affiliated Hospital of Wenzhou Medical University, Wenzhou, 325000 Zhejiang China; 7 Department of Thoracic Surgery, The First Affiliated Hospital of Shihezi University Medical College, Shihezi, 832000 Xinjiang China

**Keywords:** Immunotherapy, Tumor microenvironment, Non-small cell lung cancer, Single cell, Neutrophil

## Abstract

**Background:**

Immunotherapy has revolutionized cancer treatment, but most patients are refractory to immunotherapy or acquire resistance, with the underlying mechanisms remaining to be explored.

**Methods:**

We characterized the transcriptomes of ~92,000 single cells from 3 pre-treatment and 12 post-treatment patients with non-small cell lung cancer (NSCLC) who received neoadjuvant PD-1 blockade combined with chemotherapy. The 12 post-treatment samples were categorized into two groups based on pathologic response: major pathologic response (MPR; *n* = 4) and non-MPR (NMPR; *n* = 8).

**Results:**

Distinct therapy-induced cancer cell transcriptomes were associated with clinical response. Cancer cells from MPR patients exhibited a signature of activated antigen presentation via major histocompatibility complex class II (MHC-II). Further, the transcriptional signatures of FCRL4+FCRL5+ memory B cells and CD16+CX3CR1+ monocytes were enriched in MPR patients and are predictors of immunotherapy response. Cancer cells from NMPR patients exhibited overexpression of estrogen metabolism enzymes and elevated serum estradiol. In all patients, therapy promoted expansion and activation of cytotoxic T cells and CD16+ NK cells, reduction of immunosuppressive Tregs, and activation of memory CD8+T cells into an effector phenotype. Tissue-resident macrophages were expanded after therapy, and tumor-associated macrophages (TAMs) were remodeled into a neutral instead of an anti-tumor phenotype. We revealed the heterogeneity of neutrophils during immunotherapy and identified an aged CCL3+ neutrophil subset was decreased in MPR patients. The aged CCL3+ neutrophils were predicted to interact with SPP1+ TAMs through a positive feedback loop to contribute to a poor therapy response.

**Conclusions:**

Neoadjuvant PD-1 blockade combined with chemotherapy led to distinct NSCLC tumor microenvironment transcriptomes that correlated with therapy response. Although limited by a small patient sample size subjected to combination therapy, this study provides novel biomarkers to predict therapy response and suggests potential strategies to overcome immunotherapy resistance.

**Supplementary Information:**

The online version contains supplementary material available at 10.1186/s13073-023-01164-9.

## Background

Lung cancer is the leading cause of cancer-related death worldwide [1], with non-small cell lung cancer (NSCLC) representing approximately 85% of lung cancer cases [2]. Cancer immunotherapy through immune check point blockade (ICB) is the first-line treatment for advanced NSCLC without an identified driver-gene mutation [3]. For resectable NSCLC, immunotherapy prior to surgery (neoadjuvant immunotherapy) is emerging as a promising therapeutic regimen [4]. A common measure of neoadjuvant immunotherapy efficacy is the “major pathologic response” (MPR), which is defined as having no more than 10% residual viable tumor cells by routine hematoxylin and eosin (H&E) staining after therapy [5]. Despite the benefits of immunotherapy, its efficacy by this measure is limited. The mean MPR rate of neoadjuvant anti-PD-1/PD-L1 immunotherapy is approximately 32% (range 18 to 63%) [6]. Most patients are refractory to therapy or acquire resistance, and the underlying mechanisms remain to be explored.

The tumor microenvironment (TME) plays an important role in tumor development, progression, metastasis, and drug resistance [7]. Immunotherapy remodels the TME, and the TME in turn influences the response to immunotherapy [8]. Previous studies have characterized TME remodeling after ICB and associated the changes with clinical outcomes. ICB treatment overcomes T cell dysfunction or exhaustion and promotes clonal expansion of T cells [9]. Patients with clonotypic expansion of T cells respond best to ICB therapy [10]. Studies also suggest that the abundance of CD8+TCF7+ T cells and EOMES+CD69+CD45RO+ T cells in the TME before treatment predicts ICB response and better survival [11, 12]. Other studies have indicated that the formation of tertiary lymphoid structures (TLS), which are aggregations of B cells, T cells, dendritic cells (DC), and high endothelial venules, promote immunotherapy response [13]. The myeloid component also has been reported to be associated with immunotherapy response, with a subset of CD73+ macrophage persistence marking therapy resistance [14]. Most previous studies of immunotherapy and the TME focus on easily accessible cancer types such as melanoma. A recent study focusing on mutation-associated neoantigen (MANA) specific CD8+ T cells in NSCLC reported that these cells had hallmark transcriptional programs of resident memory T (Trm) cells during immunotherapy [15]. However, this study did not address the response of the entire TME in immunotherapy for NSCLC.

The dynamics of cancer cells and other immune cells, and a more comprehensive characterization of the TME of NSCLC during ICB treatment, is of interest for more accurately predicting patient response and providing novel therapeutic targets. Also of interest is characterization of immunotherapy in combination with other drugs, which is increasingly common in clinical use. To explore mechanisms of immunotherapy resistance and their relation to changes in the TME after PD-1 blockade combined with chemotherapy, we performed single-cell RNA sequencing (scRNA-seq), bulk RNA-seq, and non-targeted metabolomics from NSCLC samples.

## Methods

### Patient cohorts

Treatment-naïve patients with resectable NSCLC with EGFR/ALK mutation negative were enrolled in this study from September 2019 to May 2021 in our center. The patients received 2–4 cycles (3 weeks per cycle) of neoadjuvant therapy (PD-1 antibody + platinum-based chemotherapy), and then underwent surgery. In total, 39 patients were enrolled in this study, including scRNA-seq cohort (*n* = 15), independent RNA-seq cohort (*n* = 21), and 3 additional patients who only donated peripheral blood samples. The clinical information of all patients was shown in Additional file 1: Table S1.

### Sample collection

**Fig. 1:**
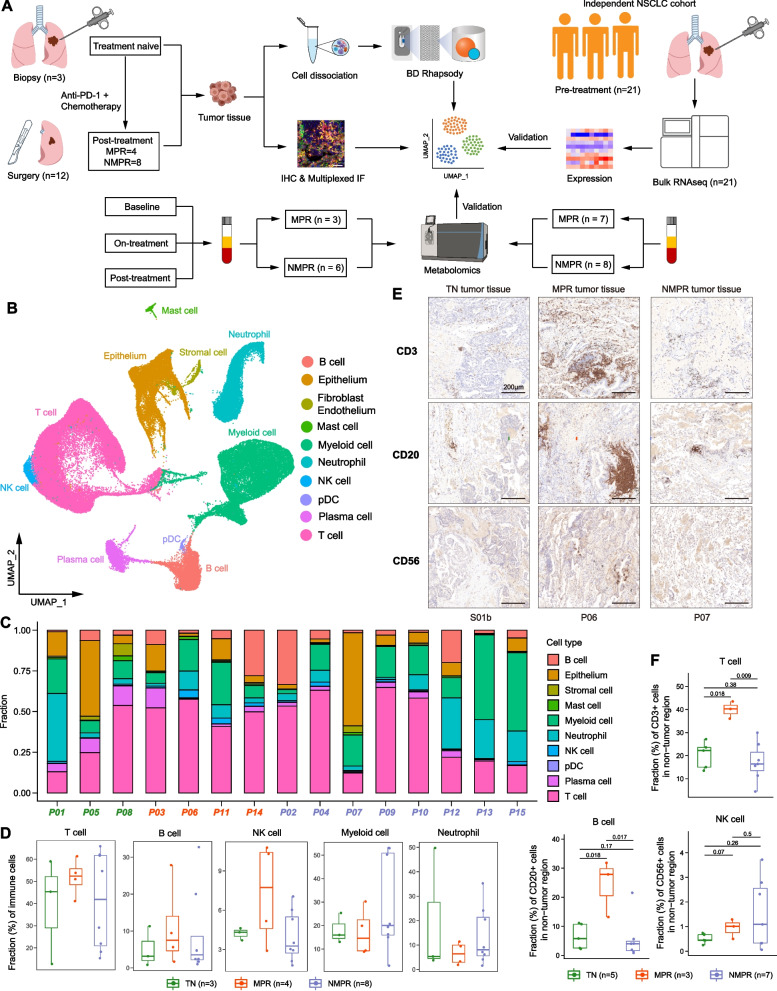
scRNA-seq analysis of NSCLC during therapy. **A** Scheme of the overall study design. **B** Uniform manifold approximation and projection (UMAP) plot of all cells colored by major cell types according to canonical markers. **C** Bar plots indicating the proportion of major cell lineages in each patient. **D** Boxplot showing cellular fractions of T, natural killer (NK), B, myeloid cells and neutrophils in TN (*n* = 3), MPR (*n* = 4), and NMPR (*n* = 8) patients. Center line indicates the median value, lower and upper hinges represent the 25th and 75th percentiles, respectively, and whiskers denote 1.5× interquartile range. Each dot corresponds to one sample. All adjusted *P* values were larger than 0.05. One-sided unpaired Wilcoxon test was used, and the *P* values were adjusted by the FDR method. **E** Representative images of immunohistochemistry (IHC) staining of canonical surface markers for T (CD3), NK (CD56), and B (CD20) cells in a TN (S01b), MPR (P06), and NMPR (P07) patient, respectively. **F** Quantification of fractions of T, NK, and B cells from the IHC images. One-sided unpaired Wilcoxon test was used

Informed consent was obtained prior to tissue acquisition, blood collection, and genomic sequencing for each patient. We obtained the primary tumor tissue by percutaneous pulmonary biopsy, bronchoscopy biopsy, or endobronchial ultrasound (EBUS) biopsy before drug administration (Additional file 2: Fig. S1A). After the last cycle of neoadjuvant therapy, the fresh tumor tissues were collected immediately by surgical resection. We collected 15 tumor samples from 15 patients for scRNA-seq and 21 pre-treatment tumor samples from 21 patients for bulk RNA-seq (Fig. 1A).

We collected 67 peripheral blood samples from 24 patients at 3 timepoints: baseline (*n* = 24), on-treatment (at the first or second cycle, *n* = 24), and post-treatment (after the last cycle, *n* = 19) (Additional file 2: Fig. S1A). Of all the 24 patients, 9 patients were from the scRNA-seq cohort and 12 patients were from the independent RNA-seq cohort. Peripheral blood samples (5 ml) were collected and stored in tubes containing EDTA. Serum was isolated by centrifugation and stored in −80°C refrigerators until being used for non-targeted metabolomics.

### Tissue dissociation and cell purification

Fresh samples from biopsy or surgery were isolated and transported rapidly to the research facility. Tissues were transported in a sterile culture dish with 10 ml 1× Dulbecco’s phosphate-buffered saline (DPBS; Thermo Fisher, Cat. no. 14190144) on ice (4 °C) to remove the residual tissue storage solution, then minced into 1–3 mm^3^ pieces in another culture dish. We used 10 mg type I collagenase (Sigma, Cat. no. C0130) dissolved in 10 ml RPMI 1640 medium (Thermo Fisher, Cat. no. 10-040-CM) with 10% fetal bovine serum (FBS; Thermo Fisher, Cat. no. SV30087.02) to digest the tissues. Tissues were dissociated at 37 °C with a shaking speed of 50 r.p.m. Cell suspensions were filtered using a 70-um nylon cell strainer and red blood cells were removed by 1× Red Blood Cell Lysis Solution (Thermo Fisher, Cat. no. 00-4333-57). Dissociated cells were washed with 1× DPBS containing 2% FBS. Cells were stained with 0.4% Trypan blue (Thermo Fisher, Cat. no. 14190144) to check the viability on Countess® II Automated Cell Counter (Thermo Fisher).

### Single-cell RNA sequencing

To capture single-cell transcriptomic information of lung cancer samples, we used the BD Rhapsody Single-Cell Analysis System (BD Biosciences) according to the manufacturer’s protocol (supported by Shanghai Biotechnology Corporation). Single-cell capture was achieved by the random distribution of a single-cell suspension across ~200,000 microwells. Beads with unique molecular identifiers (UMIs) and cell barcodes were loaded close to saturation, so that each cell was paired with a bead in a microwell. After exposure to cell lysis buffer, polyadenylated RNA molecules hybridized to the beads. Beads were retrieved into a single tube for reverse transcription. On cDNA synthesis, each cDNA molecule was tagged on the 5′ end (that is, the 3′ end of a messenger RNA transcript) with UMI and cell label indicating its cell of origin. Whole transcriptome libraries were prepared using the BD Resolve single-cell whole transcriptome amplification workflow. Briefly, Rhapsody beads were then subject to second-strand cDNA synthesis, adaptor ligation, and universal amplification. Sequencing libraries were prepared using random priming PCR of the whole transcriptome amplification products to enrich the 3′ end of the transcripts linked with the cell label and UMI. Sequencing libraries were quantified using a High Sensitivity DNA Chip (Agilent) on a Bioanalyzer 2100 and the Qubit High Sensitivity DNA Assay (Thermo Fisher Scientific). The libraries were sequenced on NovaSeq6000 (Illumina) using 2×150 chemistry. The BD Rhapsody analysis pipeline was used to process raw sequencing data (FASTQ files).

### Bulk RNA sequencing

RNA was isolated from fresh frozen tissues and perform RNA-seq. Total RNA was isolated with the RNeasy Mini Kit (Qiagen). The NEBNext Ultra RNA library (New England Biolabs) was used to construct the RNA-seq libraries according to the manufacturer’s protocol. Then, the quality-checked libraries were sequenced on the Illumina Novaseq 6000 platform.

### Non-targeted metabolomics

#### Metabolite extraction

The serum samples (100 μL) were placed in the EP tubes and resuspended with prechilled 80% methanol and 0.1% formic acid by a well vortex. Then the samples were incubated on ice for 5 min and centrifuged at 15,000*g*, 4°C for 20 min. Some of the supernatant was diluted to final concentration containing 53% methanol by LC-MS-grade water. The samples were subsequently transferred to a fresh Eppendorf tube and then were centrifuged at 15,000*g*, 4°C for 20 min. Finally, the supernatant was injected into the LC-MS/MS system analysis.

#### UHPLC-MS/MS analysis

UHPLC-MS/MS analyses were performed using a Vanquish UHPLC system (Thermo Fisher, Germany) coupled with an Orbitrap Q Exactive™ HF mass spectrometer (Thermo Fisher, Germany) in Novogene Co., Ltd. (Beijing, China). Samples were injected onto a Hypesil Goldcolumn (100×2.1 mm, 1.9μm) using a 17-min linear gradient at a flow rate of 0.2 mL/min. The eluents for the positive polarity mode were eluent A (0.1% FA in Water) and eluent B (Methanol). The eluents for the negative polarity mode were eluent A (5 mM ammonium acetate, pH 9.0) and eluent B (Methanol). The solvent gradient was set as follows: 2% B, 1.5 min; 2–100% B, 12.0 min; 100% B, 14.0 min; 100-2% B, 14.1 min; 2% B, 17 min. Q Exactive™ HF mass spectrometer was operated in positive/negative polarity mode with a spray voltage of 3.2 kV, capillary temperature of 320°C, sheath gas flow rate of 40 arb, and aux gas flow rate of 10 arb.

### Immunohistochemistry

Tissues were fixed in 4% paraformaldehyde, embedded in paraffin, cut into sections, and placed on adhesion microscope slides. Sections were subjected to immunohistochemical (IHC) staining according to standard procedures. We performed the IHC by using CD3 rabbit anti-human antibody (Biolynx, BX50022), CD20 mouse anti-human antibody (Dako, M0755), and CD56 mouse anti-human antibody (Cell Signaling Technology, 3576S). The above primary antibodies were incubated at 4°C overnight followed by using the BOND™ Polymer Refine Detection Kit (Leica, DS9800) according to the manufacturer’s instructions. Whole slide scanning was performed using panoramic MIDI under a ×20 objective lens. Tumor and stroma recognition was performed using the “tissue classification” module of HALO tissue analysis software (Indica Lab), based on the tumor morphology.

### Multiplex immunofluorescence

Multiplex immunofluorescence staining was performed using PANO 4-plex IHC kit (cat 10001100100, Panovue). We performed the fluorescent dyes by using the CD20 mouse anti-human antibody (Dako, M0755), CD86 rabbit anti-human antibody (CST, 91882S), and FCRL4 rabbit anti-human antibody (Abcam, ab239076). Different above primary antibodies were applied, followed by horseradish peroxidase-conjugated secondary antibody incubation and tyramide signal amplification. The slides were microwave heat-treated after each TSA operation. Nuclei were stained with DAPI (SIGMA-ALDRICH, D9542) after all the human antigens had been labelled. To obtain multispectral images, the stained slides were scanned using the Mantra System (PerkinElmer, Waltham, Massachusetts, USA), which captures the fluorescent spectra at 20-nm wavelength intervals from 420 to 720 nm with identical exposure time; the scans were combined to build a single stack image.

### Single-cell RNA-seq data generation and quality control

scRNA-seq FASTQ files were processed using the BD Rhapsody Whole Transcriptome Analysis (WTA) Pipeline to get a unique molecular identifier (UMI) matrix for each sample. The matrix of read counts per gene per sample was further analyzed by the Seurat package (version 3.2.2) [16] in the R software (version 3.6.3). For each cell, we used four quality control (QC) measures. Cells meeting any of the following criteria were excluded: (1) < 500 expressed genes, (2) > 20% UMIs of mitochondria genes, (3) > 50% UMIs of ribosome genes, and (4) housekeeping score (defined as the sum of the UMIs of three canonical housekeeping genes: ACTB, GAPDH and MALAT1) < 1. To exclude data from droplets containing more than one cell, doublet detection and removal were performed using Scrublet (version 0.2.1) [17]. An expected doublet rate parameter of 0.025 was used, and doublet score thresholds were chosen manually to divide putative singlet and neotypic doublet modes in the score distribution. Predicted doublets were then removed from gene-barcode matrices.

### Across-sample integration

The gene expression matrices were normalized by the *NormalizeData* function with default parameters. To adjust for biological sources of variation between samples, the standard anchor-based workflow for dataset integration in Seurat was used. As a previous study [18], 3000 or 4000 (for B cell clustering) variable features for CCA (canonical correlation analysis) [19] were chosen based on stabilized variance, and integration anchors were identified using the first 20 reduced dimensions. Integration-transformed expression values were used only for dimension reduction and clustering. The original log-normalized expression values were used for all differential expression and gene set level analyses.

### Dimension reduction and unsupervised clustering

Principal components analysis (PCA) was performed on the integration-transformed expression matrix using the *RunPCA* function, and the first 15 principal components (PCs) were used in the *FindNeighbors* function. The resolution parameters of the *FindClusters* function were different for different cell types, with 0.6 for all cells, 0.4 for T and myeloid cells, and 0.3 for epithelia, B cells, and neutrophils. Uniform manifold approximation and projection (UMAP) was performed for visualization in two dimensions using the *RunUMAP* function with the same PCs and other default parameters. Major cell lineages were assigned to each cluster of cells using the abundance of canonical marker genes, and marker genes for each cluster were found using the *FindAllMarkers* function with the parameter “*min.pct = 0.25, thresh.use = 0.25*”. For an immune cluster expressing cycle cell genes, we run clustering again to split it into T, B, and myeloid lineages. Notably, when we did cell clustering, we manually removed the clusters that expressed two or more major lineage markers (such as LYZ for myeloid cells and CD3E for T cells) on UMAP plot and probably were doublets that were not recognized by Scrublet.

### CNV estimation and identification of malignant cells

To identify malignant cells from epithelia, we used the CopyKAT algorithm (version 0.1.0) [20] to estimate the copy number variations (CNVs). The stromal cells (fibroblasts and endothelia) were used as normal reference, and the parameters were default. The sum of calculated CNV for each gene per cell was defined as the CNV score of the cell.

### Differential expression analysis and gene set variation analysis (GSVA)

Differential expression analysis comparing cells from treatment exposure or response groups was performed using the *FindAllMarkers* function with the parameter “*min.pct = 0.25, thresh.use = 0.25*”.

To assign pathway activity estimates to individual cells, we applied GSVA using standard settings, as implemented in the GSVA R package (version 1.32.0), as described previously [21]. The gene set of 50 hallmark pathways we investigated (*h.all.v7.2.symbols.gmt*) was downloaded from the Gene Set Enrichment Analysis (GSEA) website (https://www.gsea-msigdb.org/gsea/index.jsp). The differential activities of pathways between groups were calculated using limma R package (version 3.42.2). Significantly disturbed pathways were identified with Benjamini-Hochberg–corrected *P* value of ≤ 0.01.

### Gene module enrichment analysis

To estimate the signature of MHC-II antigen presentation in cancer cells, we calculated the enrichment scores for each cell using the *AddModuleScore* function in Seurat with the gene list from the REACTOME_MHC_CLASS_II_ANTIGEN_PRESENTATION pathway (*c2.cp.reactome.v7.2.symbols.gmt*, download from https://www.gsea-msigdb.org/gsea/index.jsp). To explore the cytotoxic and exhausted functions of T and NK cells, we calculated the cytotoxic score and exhausted score for each cell using the canonical cytotoxic (GZMA, GZMB, GZMK, GNLY, IFNG, PRF1, and NKG7) and exhausted (LAG3, TIGIT, PCCD1, HAVCR2, CTLA4, LAYN, and ENTPD1) markers, respectively. With the same method, we used the gene list (Additional file 3: Table S2) in “LM22.xls” from CIBERSORT [22] to estimate the phenotype (M0, M1, or M2) for each macrophage. We also calculated the antigen presentation score for DCs with the previously reported markers (Additional file 3: Table S2) [23]. A mean value of module scores of a cell cluster (≥ 10 cells) from an individual sample was calculated to present the signature level.

### Cellular fraction calculation

For each sample, we calculated the cellular fraction for each major cell lineage (T, B, myeloid cells), and for the subpopulations of major cell lineages, cellular proportions were calculated by the fractions in corresponding major immune lineages. Notably, the samples that had less than 10 cells in a major lineage were removed to do downstream statistic test. The significance of differences among response groups for the fractions was compared using one-sided unpaired Wilcoxon rank sum test, and the *P* values were adjusted by the false discovery rate (FDR) method for multiple parallel tests.

### Trajectory analysis

#### RNA velocity

The bam files from the WTA Pipeline were converted into sam files first using samtools (version 1.7) [24]. Then the cell barcode tag “MA” for each sequence was replaced by “UB” which could be recognized by the *velocyto run* function from RNA velocity (version 0.17.17) algorithm [25]. We removed the sequences without cell barcode, and the sam files were converted back into bam files and sorted using samtools. The sorted bam files were used to generate loom files using the *velocyto run* function with the genome annotation file “GRCh38.gtf”. The loom files for each sample were merged into one loom file. To calculate the velocity and visualize on plot, we used the scVelo (version 0.2.3) [26] method using steady-state mode, following the Seurat to RNA velocity guides (https://github.com/basilkhuder/Seurat-to-RNA-Velocity).

#### Monocle2

We also used Monocle2 (version 2.14.0) [27] to infer the cell lineage trajectory of T cells, myeloid cells, DCs, and neutrophils with the top 1000 signature genes with *q* value < 0.001 calculated by *differentialGeneTest* function. The differentiation trajectory was inferred with the default parameters of Monocle after dimension reduction and cell ordering.

### Cell-cell interaction analysis

We used CellPhoneDB (version 2.1.5) [28] to infer cell-cell interaction between different cell types. This method infers the potential interaction strength between two cell subsets based on gene expression level and provides the significance through permutation test (1000 times). The enriched ligand-receptor interactions between two cell subsets were calculated based on the permutation test. We extracted significant ligand-receptor pairs with *P* value < 0.01.

### NicheNet analysis

NicheNet (version 1.0.0) [29], a powerful tool that predicts ligands driving the transcriptomic changes of target cells, was used to identify potential ligands that drive the unique phenotype of B cell and neutrophil subsets. As described previously [30], we used all expressed genes of the B4_FCRL4 cells as the background of genes and the top 50 genes ordered by log2FC as gene sets of interest. Genes were considered as expressed when they have nonzero values in at least 10% of the cells in a cell type. We only used the expressed receptors in B4_FCRL4 cells to construct the expressed ligand-receptor interactions and calculate the ligand activity using the *predict_ligand_activities* function. For the Neu_CCL3 and Neu_IFIT3 cells, the same analyses were performed.

### SCENIC analysis

As descripted [30], activated regulons in each neutrophil subset were analyzed using SCENIC [31] with raw count matrix as input. The co-expression network was calculated by *runGenie3* and the regulons were identified by *RcisTarget*. The regulon activity for each cell was scored by *AUCell*.

### Bulk RNA-seq data processing and quantification

Raw FASTQ files were aligned on the hg38 genome reference using the STAR aligner (version 2.7.4a) [32] with default parameters. Salmon (version 1.3.0) [33] was used to quantitate gene expression by transcripts per kilobase million (TPM). The TPM matrix was transformed by log2(TPM+1).

### Gene signature estimation in bulk RNA-seq

The cell signatures of B4_FCRL4 and Mono_CX3CR1 were estimated by ssGSEA method in the GSVA R package (version 1.32.0). The ssGSEA transforms specific gene expression patterns into quantities of cell populations in individual tumor samples at the bulk level. The markers of the two cell types (Additional file 3: Table S2) were used as the gene sets in the *gsva* function with the parameters “*method=‘ssgsea’, kcdf=‘Gaussian’, abs.ranking=TRUE*”. For melanoma cohort 2 [34], we analyzed the prognostic value of the two cell signatures. The patients were divided into high and low signature groups by the median value, and the Kaplan–Meier survival curves with the cumulative number of events table and log-rank test were plotted by survminer (version 0.4.8) and survival (version 3.1-8) R package.

### TCGA analysis

The analyses of lung adenocarcinoma (LUAD) from the TCGA database were performed on the TCGA visualization web server, GEPIA2 (http://gepia2.cancer-pku.cn/), developed by Zeming Zhang [35]. The server provided several function modules, and we used the “Survival Analysis” module to explore the correlation between the expression of genes of interest and overall survival. The patients were divided into high and low signature groups and hazards ratio (HR) was calculated. The apoptosis signature was calculated by ssGSEA method by the gene list of apoptosis from KEGG_APOPTOSIS pathway (*c2.cp.kegg.v7.2.symbols.gmt*, download from https://www.gsea-msigdb.org/gsea/index.jsp). The signatures of Macro_SPP1 and Neu_CCL3 were estimated by the marker genes of the two cell types (Additional file 3: Table S2).

### Metabolomic data processing and metabolite identification

The raw data files generated by UHPLC-MS/MS were processed using the Compound Discoverer 3.1 (CD3.1, Thermo Fisher) to perform peak alignment, peak picking, and quantitation for each metabolite. The main parameters were set as follows: retention time tolerance, 0.2 min; actual mass tolerance, 5ppm; signal intensity tolerance, 30%; signal/noise ratio, 3; and minimum intensity, etc. After that, peak intensities were normalized to the total spectral intensity. The normalized data was used to predict the molecular formula based on additive ions, molecular ion peaks, and fragment ions. Then peaks were matched with the mzCloud (https://www.mzcloud.org/), mzVault, and MassList database to obtain the accurate qualitative and relative quantitative results. When data were not normally distributed, normal transformations were attempted using of area normalization method.

### Drug sensitivity analysis

The gene expression data (RPKM matrix) of NSCLC cell lines was downloaded from the (CCLE) database (https://portals.broadinstitute.org/ccle/), and the IC50 data of drugs was downloaded from the Genomics of Drug Sensitivity in Cancer (GDSC) database (www.cancerRxgene.org). The gene expression of 65 NSCLC cell lines and the IC50 data of 16 drugs were used to analyze in this study. We first estimated the signature of the 5 AKR family genes (AKR1C1-3 and AKR1B1/10) for each cell line using ssGSEA method, then we calculated the Pearson correlation coefficient between the signature and IC50 value for each drug.

### Statistics

All statistical analyses and presentations were performed using R software (version 3.6.3). All statistical tests used are defined in the figure legends. Statistical significance was set at *P* or adjusted *P* < 0.05.

## Results

### scRNA-seq analysis of NSCLC during PD1 blockade combined with chemotherapy

We prospectively collected fresh tumor samples from a total of 15 patients with clinical stage IIIA NSCLC for analysis by scRNA-seq (Fig. 1A, Additional file 2: Fig. S1A and Additional file 1: Table S1). For three patients, samples were collected by biopsy before treatment and classified as treatment naïve (TN; *n* = 3). For the remaining 12 patients, samples were taken from surgical resections after PD-1 antibody combined with chemotherapy treatment. The 12 post-treatment samples were categorized into two groups: MPR (*n* = 4) and NMPR (non-major pathologic response; *n* = 8) based on pathologic assessment [5]. The dataset analyzed here also includes bulk RNA-seq from fresh biopsies from 21 independent TN patients (Additional file 1: Table S1).

The fresh tissues were rapidly digested to a single-cell suspension, and all single-cell transcriptomes were generated using commercial BD Rhapsody platform. After quality control and removal of doublets, transcriptomes from 92,330 cells with a median of 1256 genes per cell were used for further analyses. To mitigate batch effects from patients (Additional file 2: Fig. S1B) and allow for joint analysis of malignant and non-malignant cells, we performed canonical correlation analysis (CCA) and aggregated cells from different patient samples. Unsupervised clustering of all cells identified 26 clusters (Fig. 1B and Additional file 2: Fig. S1C), with no significant batch effects observed across different patients, PD-1 antibodies, or response groups (Additional file 2: Fig. S1D-E). Further, the average gene numbers and unique molecular identifiers (UMIs) were comparable between different clusters (Additional file 2: Fig. S1F). We then annotated the 26 clusters into T cells, NK cells, B cells, myeloid cells, neutrophils, plasma cells, plasmacytoid DC (pDC), mast cells, stromal cells (fibroblasts/endothelia), and epithelial cells, according to the expression of corresponding canonical marker genes (Fig. 1B and Additional file 2: Fig. S1G).

To characterize the TME remodeling in response to treatment, we calculated the fraction of different cell types in TN, MPR, and NMPR patients (Fig. 1C, D). We observed that the fraction of T cells, NK cells, and B cells were increased in MPR patients, although we did not get positive *P* values due to limited sample sizes (Fig. 1D). To further validate this, we performed immunohistochemical (IHC) staining in 10 post-treatment surgical tumor tissues (3 MPR and 7 NMPR, corresponding to scRNA-seq samples) and another 5 treatment-naïve surgical tumor tissues (Additional file 1: Table S1). IHC staining verified the increased abundance of T (CD3+) and B (CD20+) cells in MPR samples, except NK (CD56+) cells (Fig. 1E, F, and Additional file 2: Fig. S2). This was consistent with previous reports that T and B cell expansion was associated with better response to ICB [10, 13]. Myeloid cells were enriched in the TME, but showed no obvious differences among TN, MPR, and NMPR patients (Fig. 1D). However, myeloid cells are known to have diverse and complex functions in the TME [36], which are further explored later in this study. We also identified 5–20% cells as neutrophils in the TME of NSCLC [37], which are usually absent in previous scRNA-seq studies using 10X Genomics, reflecting the advantage of BD Rhapsody in capturing neutrophils.

### Increase of normal lung epithelial cells and detection of residual cancer cells in pCR patients after combined therapy

**Fig. 2:**
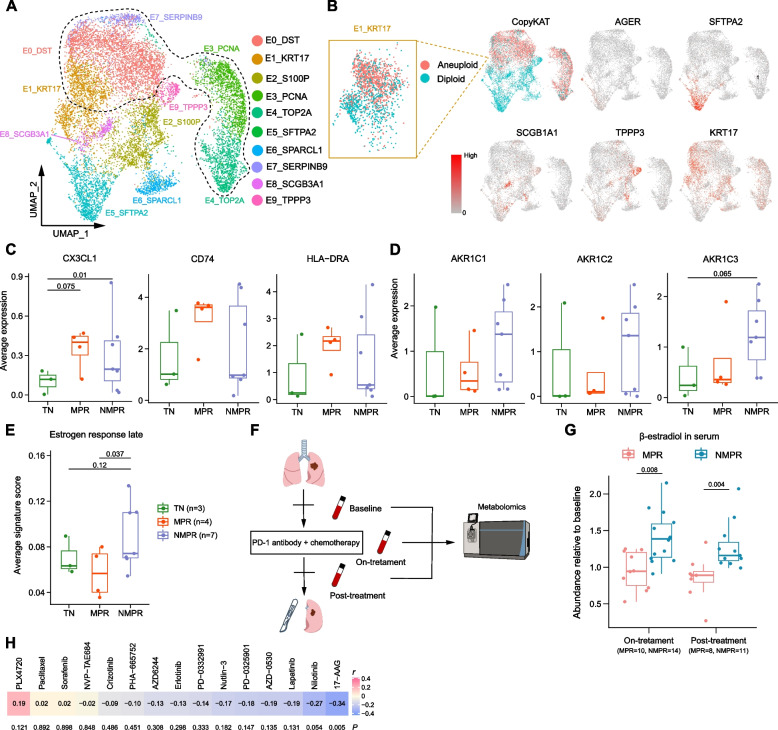
Epithelial cell reprograming after therapy. **A** UMAP plot of epithelia colored by clusters. The cells within the black dash line were malignant cells based on copy number variations (CNVs) inferred by the CopyKAT algorithm. **B** UMAP plots of epithelia colored by CopyKAT and normal lung epithelial markers. In the left top panel, the cells in red were predicted to be malignant cells and blue were normal cells. The cells in cluster E1_KRT17 contained both malignant and normal cells. **C** Boxplots of the average expression of CX3CL1, CD74, and HLA-DRA in malignant cells in TN (*n* = 3), MPR (*n* = 4), and NMPR (*n* = 7, one sample with less than 10 malignant cells was removed) patients. Center line indicates the median, lower, and upper hinges represent the 25th and 75th percentiles, respectively, and whiskers denote 1.5× interquartile range. One-sided *t*-test was used, and the *P* values were adjusted by the FDR method. **D** Boxplots of the average expression of ARK1C1-3 in malignant cells in TN (*n* = 3), MPR (*n* = 4), and NMPR (*n* = 7) patients. One-sided *t*-test was used, and the *P* values were adjusted by the FDR method. **E** Boxplot of the average signature score in malignant cells in TN (*n* = 3), MPR (*n* = 4), and NMPR (*n* = 7) patients. One-sided *t*-test was used. **F** Longitudinal serums were collected from 24 patients (10 were assessed as MPR, and 14 as NMPR after surgery) at baseline, on-treatment and post-treatment timepoint. Non-targeted Metabolomic was conducted to detect the abundance of β-estradiol. **G** Boxplots of the β-estradiol abundance relative to baseline in 24 patients (10 patients were assessed as MPR and 14 as NMPR after surgery) at on-treatment and post-treatment timepoint. Two-sided unpaired Wilcoxon test was used. **H** Correlation analysis between the signature of AKR family genes and IC50 values in NSCLC cell lines under the condition of different drugs. *P* values were determined by two-sided Pearson correlation test

We next investigated populations of epithelial cells. We first re-clustered the epithelial cells into 10 populations and separated malignant and normal cells using the CopyKAT algorithm [20] based on copy number variations (CNVs) (Fig. 2A, B and Additional file 2: Fig. S3A-B). Clusters E0_DST, E3_PCNA, E4_TOP2A, E7_SERPINB9, and a subpopulation of E1_KRT17 had higher CNV scores than other clusters and were inferred to be malignant cells (Fig. 2B and Additional file 2: Fig. S3C-D). The normal clusters were annotated as alveolar cells (E5_SFTPA2, type I: AGER, type II: SFTPA2), secretory club cells (E8_SCGB1A1), ciliated cells (E9_TPPP3), and basal epithelial cells (subpopulation of E1_KRT17) based on traditional markers (Fig. 2A, B) [21]. The fractions of alveolar cells (E5_SFTPA2), club cells (E8_SCGB1A1), and ciliated cells (E9_TPPP3) were increased after therapy, especially for MPR patients (Additional file 2: Fig. S3E-F). This indicated that combined therapy promoted expansion of normal epithelial cells after eliminating malignant cells. The normal epithelial cells may contribute to reconstruct normal lung structure in the previous tumor bed. In addition, it also has been reported that SCGB1A1+ club cells could increase the efficacy of ICB in lung cancer by promoting infiltration of cytotoxic cells [38].

When comparing the cellular fraction of epithelial cells between different patients, we noted the enrichment of the malignant cluster E7_SERPINB9 in P06. This is unexpected, because P06 was classified as having a pathologic complete response (pCR; Additional file 2: Fig. S3E,G), which is defined by a complete absence of viable tumor cells upon H&E staining [5]. Although it is possible that this arose from sampling bias among the histopathologic slides, it is more likely that the sample from P06 contains malignant cells with genome alterations, but not morphological changes that can be detected by traditional histopathology. Our observation is consistent with previous reports that pCR patients may nonetheless experience tumor recurrence [39]. This reflects the presence of molecular residual disease (MRD) in pCR patients, a rising biomarker for NSCLC immunotherapy [40]. MRD is generally detected by circulating tumor DNA in serum. This case suggests that scRNA-seq has the ability in assessing MRD, which may be necessary to detection even for pCR patients.

### Distinct molecular characteristics of malignant cells distinguish MPR and NMPR

To better characterize the malignant cell transcription programs activated in response to therapy, we performed differential expression analysis among TN, MPR, and NMPR patients. We were particularly interested in expression patterns that may drive interactions with the immune system and perform as signatures of therapy response. In response to therapy, malignant cells from MPR patients highly expressed CX3CL1, CD74, and major histocompatibility complex class II (MHC-II) genes (Fig. 2C and Additional file 2: Fig. S4A). Each key component of this MPR signature is addressed in turn below.

CX3CL1 is the ligand of CX3CR1. Previous studies have reported that CX3CR1 is highly expressed in many immune cells including NK cells [41] and monocytes [42]. CX3CL1 was downregulated in lung adenocarcinoma (LUAD) tumors compared to normal lung tissues in TCGA cohorts (Additional file 2: Fig. S4B), indicating tumor immune evasion. Therefore, cancer cells expressing CX3CL1 in response to therapy may promote immune cell infiltration into the TME, thereby improving overall survival (Additional file 2: Fig. S4D).

CD74 and MHC-II genes, also components of the MPR signature, are required for tumor antigen presentation [43]. We observed that the gene signature of antigen presentation via MHC-II was higher in cancer cells from MPR patients than TN or NMPR patients (Additional file 2: Fig. S4C). Previous studies have shown that MHC-II expression is associated with anti-PD-1 therapy response [44], progression-free, and overall survival in melanoma [45]. Consistent with these observations, higher expression of CD74 and HLA-DRA was associated with a better prognosis in TCGA-LUAD cohorts (Additional file 2: Fig. S4D).

Compared to TN and MPR patients, we observed that enzymes in the Aldo-Keto Reductase family (AKR1B1/10 and AKR1C1-3) were highly expressed in cancer cells from NMPR patients (Fig. 2D and Additional file 2: Fig. S4E-F). The AKR1B family has been previously reported to promote tumor metastasis and drug resistance [46–48], and the AKR1C family (hydroxysteroid dehydrogenases) was involved in estrogen metabolism, catalyzing the reduction of estrone to β-estradiol [49]. Consistent with this, Gene Set Variation Analyses (GSVA) revealed that following combined therapy, estrogen response pathways were upregulated in malignant cells from NMPR patients (Fig. 2E and Additional file 2: Fig. S4G). Only one of the eight (12.5%) NMPR patients was female, while two of the four (50%) patients in MPR group were female (Additional file 1: Table S1). None of the 15 patients used any estrogen-related drugs during therapy. Thus, the data suggest that estrogen metabolism may be aberrantly high in NMPR patients following treatment.

To validate this, we used non-targeted metabolomics to detect the abundance of steroids in serum from cells collected at baseline (before neoadjuvant therapy), on-treatment (at the first or second cycle, 3 weeks per cycle, total 2-4 cycle) and post-treatment (4 weeks after the last drug administration, blood samples were collected before surgery) in 10 MPR (30% female) and 14 NMPR (7% female) patients (Fig. 2F and Additional file 1: Table S1). In confirmation of the previous result, levels of β-estradiol were significantly elevated in NMPR patients compared to baseline during therapy (Fig. 2G, Additional file 2: Fig. S4H and Additional file 4: Table S3). When removing the patients from scRNA-seq cohort, the results were similar (Additional file 2: Fig. S4I). Thus, elevated estrogen levels in serum could reflect poor response to immunotherapy. Estradiol has been reported to be an immunosuppressor in the TME [50], through promoting the infiltration of M2 macrophages [51], mobilization of myeloid-derived suppressor cells (MDSCs) [52], and expansion of Tregs [53]. These suggested that estradiol may generate an immunosuppressive TME in the NMPR patients.

To identify potential drugs that may be effective on cancer cells in NMPR patients, we explored data in NSCLC cell lines from the Genomics of Drug Sensitivity in Cancer (GDSC) database. We found that the NMPR signature was negatively correlated with the IC50 (half the maximal inhibitory concentration) of 17-AAG (Fig. 2H), an inhibitor of HSP90, suggesting that cancer cells in NMPR patients may be sensitive to 17-AAG. Notably, 17-AAG is reported to inhibit estrogen signaling by disrupting HSP90 [54].

### The degree of cytotoxic T/NK cell expansion and reduction of suppressive Tregs after combined therapy is positively associated with pathologic response

**Fig. 3:**
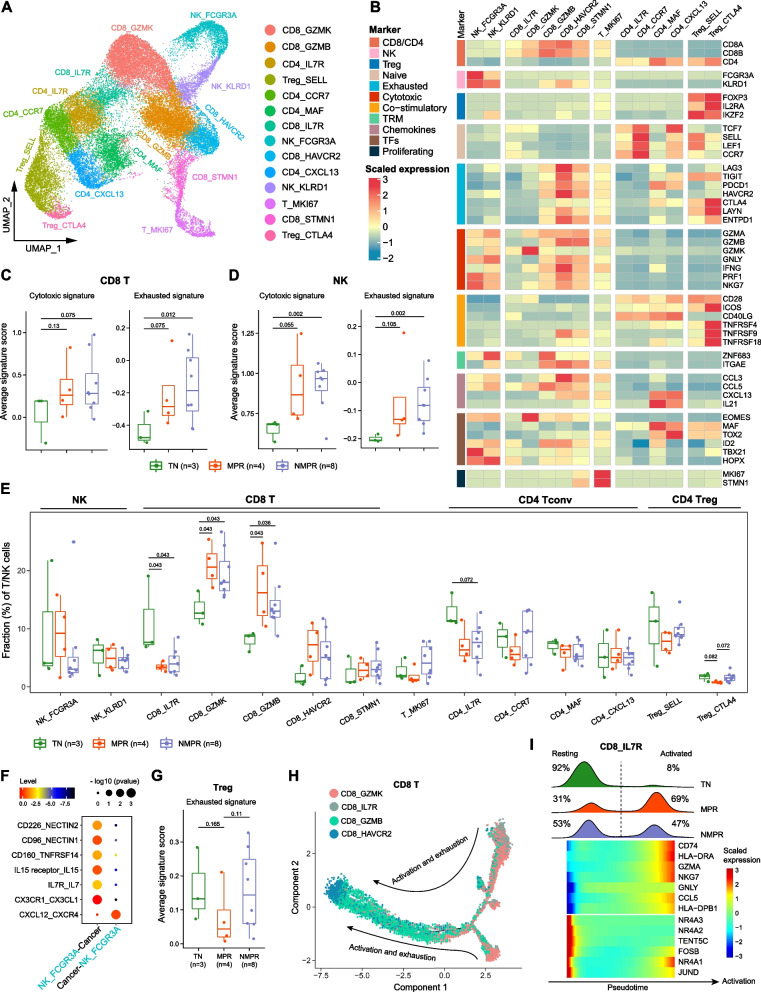
T/NK cell remodeling after therapy. **A** UMAP plot of T/NK cells colored by clusters. **B** Heatmap of normalized expression of canonical T/NK cell marker genes among clusters. TRM, tissue-resident memory. **C** Boxplots of the average cytotoxic and exhausted signature scores for CD8+ T cells in TN (*n* = 3), MPR (*n* = 4), and NMPR (*n* = 8) patients. Center line indicates the median, lower, and upper hinges represent the 25th and 75th percentiles, respectively, and whiskers denote 1.5× interquartile range. One-sided *t*-test was used. **D** Boxplots of the average cytotoxic and exhausted signature scores for NK cells in TN (*n* = 3), MPR (*n* = 4), and NMPR (*n* = 8) patients. One-sided *t*-test was used. **E** Boxplot showing cellular fractions of each T/NK cluster in TN (*n* = 3), MPR (*n* = 4), and NMPR (*n* = 8) patients. All differences with adjusted *P* < 0.10 are indicated. One-sided unpaired Wilcoxon test was used and the *P* values were adjusted by the FDR method. **F** Summary of selected ligand-receptor interactions from CellPhoneDB between cancer cells and CD16+ NK cells in MPR patients. **G** Boxplots of the average exhausted signature scores for Tregs in TN (*n* = 3), MPR (*n* = 4), and NMPR (*n* = 8) patients. One-sided *t*-test was used. **H** The developmental trajectory of CD8+ T cells inferred by Monocle2. The memory CD8+ T cells (CD8_IL7R) and effector memory (CD8_GZMK) T cells were the roots of differentiation, and the exhausted CD8+ T cells (CD8_HAVCR2) were in the end-point state. **I** Heatmap of the top differential genes in memory (CD8_IL7R) cells along the pseudo-time (lower panel). The distribution of CD8_IL7R cells during the transition (divided into 2 phases: resting and activated) in TN, MPR, and NMPR patients, along with the pseudo-time (upper panel)

Next, we explored the dynamics of immune cell lineages in the TME in response to therapy. Since T cells are the most abundant tumor-infiltrating lymphocytes in the TME, we re-clustered T/NK cells and identified 14 clusters (Fig. 3A, B and Additional file 2: Fig. S5A). These includes 2 subtypes of NK cells (NK_FCGR3A and NK_KLRD1), 5 subtypes of CD8+ T cells (CD8_IL7R, memory T [Tm]; CD8_GZMK, effector memory T [Tem]; CD8_GZMB, Trm; CD8_HAVCR2, exhausted T [Tex] and CD8_STMN1, cycling effector T), 4 subtypes of conventional CD4+ T cells (CD4_CCR7, naïve T; CD4_IL7R, memory T; CD4_MAF, mature follicular helper T [Tfhs] [55]; and CD4_CXCL13, naïve Tfhs), 2 subtypes of regulatory T (Treg) cells (Treg_SELL, naïve-like Treg; Treg_CTLA4, activated Treg), and 1 proliferating subtype (T_MKI67).

We further calculated the cytotoxic and exhausted gene signatures for CD8+ T cells and NK cells for TN, MPR, and NMPR patients. The cytotoxic and exhausted signatures were both significantly increased after therapy (Fig. 3C, D). This corresponds to an increase in the factions of all CD8+ T clusters after therapy (Fig. 3E). Relative to TN patients, the fractions of Tem (CD8_GZMK) and Trm (CD8_GZMB) and cycling effector T cells (CD8_STMN1) were increased in post-treatment samples, with the increase more pronounced in MPR patients (Fig. 3E). The CD8_GZMK cells can be resident in the TME and then locally expand after ICB, or newly infiltrate from peripheral blood [56]. The increase of Trm (CD8_GZMB) was consistent with expansion of neoantigen-specific T cells in NSCLC after immunotherapy [15].

We observed a higher fraction of Tex (CD8_HAVCR2) in both MPR and NMPR patients relative to TN patients following combined therapy. Recent studies have reported that exhausted T cells are specifically derived from tumor-specific T cells [57], and an increase in exhausted-like T cells is associated with ICB response [58]. To determine the source of exhausted T cells, we performed differential expression analysis before and after therapy in exhausted T cells (Additional file 2: Fig.S5B). We found that the transcription factors (TFs), NR4A2-3, that are associated with T cell exhaustion [59] were enriched in TN patients. This indicates that the T cells may have been exhausted before treatment, driven by NR4A2/3 during chronic T cell dysfunction. Cytotoxic (GZMH, NKG7, and PRF1) and exhausted markers (LAG3 and TIGIT) were both highly expressed in post-treatment patients. Tex cells that remain after treatment may arise from the coupled activation, expansion, and exhaustion process for cytotoxic T cells, which has been reported to be more evident in responders [18].

Cluster NK_FCGR3A was most representative of cytotoxic cells and was distinguished from NK_KLRD1 cells by expression of FCGR3A (CD16a), FGFBP2, and CX3CR1 (Fig. 3B) [41]. Given the expression of CX3CL1 in cancer cells from MPR patients (Fig. 2C), it was possible that NK_FCGR3A cells were recruited into the TME by CX3CL1. As expected, cell-cell interaction analysis using the CellPhoneDB algorithm [28] showed the CX3CL1-CX3CR1 interaction between cancer cells and NK_FCGR3A cells was significantly enriched in MPR patients (Fig. 3F).

We next focused on Tregs. Activated Tregs have been previously reported to have a stronger immunosuppressive function than naïve Tregs, and to be correlated with poor prognosis [60]. Consistently, activated Tregs (Treg_CTLA4, expressing TNFRSF4 and TNFRSF9) decreased only in MPR patients. The proportion of naïve Tregs (Treg_SELL, expressing SELL and LEF1) decreased in both MPR and NMPR patients relative to TN patients (Fig. 3E). MPR patients consistently showed lower Treg exhausted signature than NMPR patients (Fig. 3G).

Our analysis revealed the expansion and activation of cytotoxic T cells and CD16+ NK cells, and reduction of immunosuppressive Tregs after treatment. The strength of these trends was associated with positive response to combined therapy.

### Therapy promotes the differentiation of memory T cells into an effector phenotype

After combined therapy, memory CD8+ T cells (CD8_IL7R) decreased while effector T cells increased (Fig. 3E). This suggested that treatment might directly induce the activation of memory T cells into a cytotoxic phenotype. To test this hypothesis, we performed trajectory analysis using Monocle2 [27]. One detected transition path went from Tem (CD8_GZMK) to Trm (CD8_GZMB) to Tex cells (CD8_HAVCR2; Fig. 3H). This path confirmed the sequential activation and exhaustion of CD8+ T cells in the TME. The analysis also showed that the cytotoxic cells may differentiate directly from Tm cells (CD8_IL7R). Two origins of cytotoxic T cells were confirmed using RNA velocity algorithm (Additional file 2: Fig. S5C), another trajectory analysis algorithm [25]. When we delineated the distribution of the CD8_IL7R cells in pseuso-time, we noticed that the CD8_IL7R cells could be categorized into 2 phases (Fig. 3I). The CD8_IL7R cells in resting phase highly expressed NR4A1-3, while cytotoxic-related genes (GZMA, NKG7, and CCL5) and MHC-II genes (CD74 and HLA-DRA) were upregulated in the activated phase (Fig. 3I). The proportion of activated cells was increased after therapy, and more CD8_IL7R cells were activated in MPR than NMPR samples (Fig. 3I and Additional file 2: Fig. S5D). Combined, these observations suggest that therapy could activate memory CD8+T cells into an effector phenotype, and the activation was most pronounced in MPR patients.

### FCRL4+FCRL5+ memory B cells predict response to ICB and boost immunotherapy through activating CD4+ T cells

**Fig. 4:**
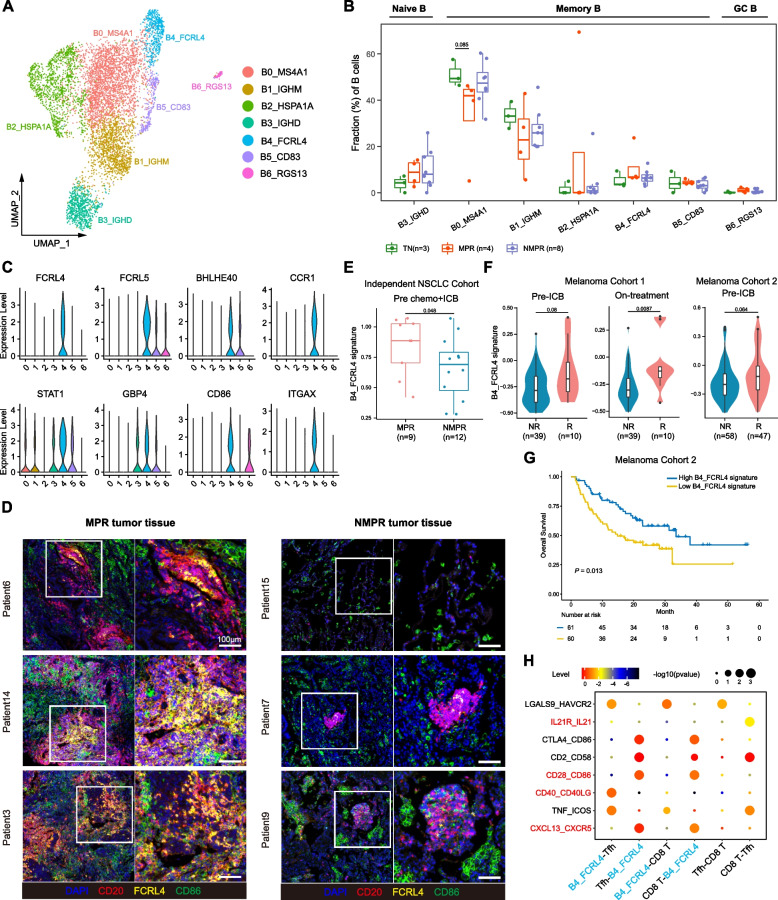
B cell remodeling after therapy. **A** UMAP plot of B cells colored by clusters. **B** Boxplot showing cellular fractions of each B cluster in TN (*n* = 3), MPR (*n* = 4), and NMPR (*n* = 8) patients. Center line indicates the median, lower, and upper hinges represent the 25th and 75th percentiles, respectively, and whiskers denote 1.5× interquartile range. All differences with adjusted *P* < 0.10 are indicated. One-sided unpaired Wilcoxon test was used, and the *P* values were adjusted by the FDR method. **C** Violin plots of marker genes of B4_FCRL4 cells across clusters. **D** In situ multiplex immunofluorescence images of B4_FCRL4 cells in MPR and NMPR tumor tissues. **E** Violin and box plots of B4_FCRL4 signature in our validation cohort (9 patients were assessed as MPR and 12 as NMPR after surgery) before ICB + chemotherapy. One-sided unpaired Wilcoxon test was used. **F** Violin and box plots of B4_FCRL4 signature in responders (R) and non-responders (NR, removing SD patients) in advanced melanoma cohorts. Two-sided unpaired Wilcoxon test was used. **G** Kaplan–Meier survival curves of the signature of B4_FCRL4 in advanced melanoma cohort 2. Survival curves were compared by the log-rank test. **H** Summary of selected ligand-receptor interactions from CellPhoneDB among B4_FCRL4 cells, Tfhs and CD8+ T cells

Studies indicate that B cells are actively involved in anti-tumor immunity after neoadjuvant chemotherapy [61]. To assess the B cell diversity after therapy, we re-clustered B cells into 7 subclusters (Fig. 4A and Additional file 2: Fig. S6A-B), including 5 subgroups of memory B cells (CD27+GPR183+IGHD-, B0_MS4A1, B1_IGHM, B2_HSP1A1, B4_FCRL4 and B5_CD83), 1 naïve B cell (CD27-IGHD+, B3_IGHD), and 1 germinal center (GC) B cell (B6_RGS13).

To characterize the function of different B cell subsets, we compared their cell-type fractions in TN, MPR, and NMPR patients. Naïve B cells were increased after treatment, while memory B cells were slightly reduced (Additional file 2: Fig. S6C). Although memory B cells in general decreased following combined treatment, the FCRL4+FCRL5+ B cells (B4_FCRL4), defined as “atypical memory B cells” [62], exhibited a slightly increasing trend in MPR patients (Fig. 4B). The FCRL4 and FCRL5 genes encode the Fc receptors for IgA and IgG, respectively, and are drivers of human memory B cell activations [62]. B cells expressing FCRL4 have been previously reported to be associated with inflammation in rheumatoid arthritis [63] and viral infections [64]. Among TCGA-LUAD patients, we consistently found that patients with high expression of FCRL4 and FCRL5 had a better prognosis (Additional file 2: Fig. S6D-E). Also highly expressed in B4_FCRL4 cells were interferon-stimulated genes (CCR1, STAT1, and GBP4), co-stimulatory molecule (CD86), and activated follicular B cell marker (BHLHE40) [65]. Consistently, immunofluorescence staining showed that FCRL4+FCRL5+ cells were much more enriched in MPR than NMPR samples (Fig. 4D and Additional file 2: Fig. S6F). Interestingly, we noticed that CD20+ B cells aggregated in TLS and FCRL4+FCRL5+ B cells located in the center of the TLS (Fig. 4D and Additional file 2: Fig. S6G). Taken together, our analysis suggests that FCRL4+FCRL5+ B cells are associated with anti-tumor activity and a positive response to combined therapy.

We investigated whether the signature from FCRL4+FCRL5+ B cells could serve as a positive biomarker for immunotherapy. We first evaluated the B4_FCRL4 gene signature (Additional file 3: Table S2) in our validation cohort. The signature scored significantly higher in MPR patients before ICB combined with chemotherapy (Fig. 4E). We then performed similar analyses on published datasets from two independent melanoma cohorts with ICB treatment [34, 66]. Although the melanoma cohorts were not in the neoadjuvant setting, the B4_FCRL4 signature also perform well in predicting immunotherapy response. The B4_FCRL4 signature was higher in responders (complete response or partial response) than non-responders (stable disease or progressive disease) before and after therapy in both cohorts (Fig. 4F). Higher B4_FCRL4 signature was associated with improved survival in previously published “melanoma cohort 2” [34] (Fig. 4G). These results indicate that the signature of FCRL4+FCRL5+ B cells can be used as biomarker for predicting response to ICB.

To explore the underlying mechanisms for the activation and function of FCRL4+FCRL5+ B cells, we performed NicheNet analysis [29], which predicts ligands driving the transcriptomic changes of target cells. Several IFNα genes, tumor necrosis factor (TNF), and IL27 were predicted as possible ligands driving the phenotype of B4_FCRL4 cells (Additional file 2: Fig. S6H). CellPhoneDB analysis revealed that FCRL4+ FCRL5+ B cells could interact with Tfhs through ligand-receptor pairs: CXCL13-CXCR5, CD40-CD40LG, and CD28-CD86 (Fig. 4H). CXCL13-CXCR5 interaction between B cells and Tfhs is essential for the formation of TLS [67]. It has been reported that tumor-specific B cell drive activation of tumor-specific Tfhs and activated Tfhs could enhance the effector function of CD8+ T cells by secreting IL21 [55]. In our study, we also noticed that the IL21-IL21R interaction was significantly enriched between Tfhs and CD8+ T cells (Fig. 4H). The data suggest that in the TME of immunotherapy responders, FCRL4+ FCRL5+ B cells are driven by IFNα, TNF, and IL27 signals, and Tfhs are activated by these B cells to enhance anti-tumor immunity through secreting IL21.

### Patrolling monocytes were recruited by CX3CL1 and predict immunotherapy response

**Fig. 5:**
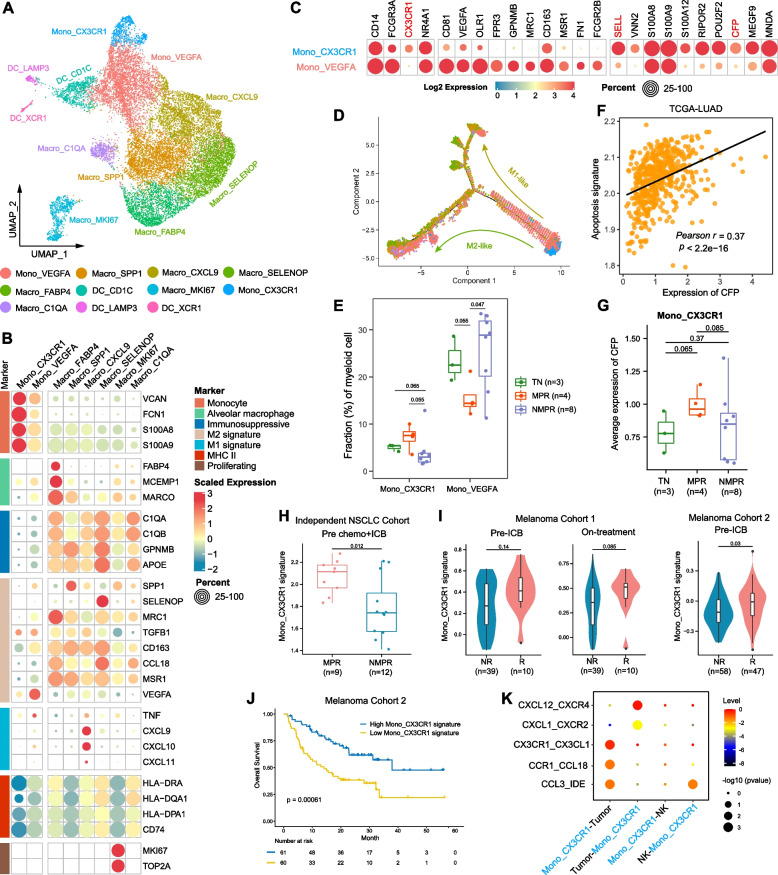
Monocyte remodeling after therapy. **A** UMAP plot of myeloid cells colored by clusters. **B** Heatmap of normalized expression of monocyte/macrophage marker genes among clusters. **C** Heatmap of selected marker genes of defined monocyte clusters. **D** The developmental trajectory of monocyte/macrophages inferred by Monocle2. The Mono_CX3CR1 cells were the roots of trajectory, and differentiated into M1-like (Macro_CXCL9) or M2-like (Macro_SELENOP and Macro_C1QA) cells. **E** Boxplot showing cellular fractions of each monocyte cluster in TN (*n* = 3), MPR (*n* = 4), and NMPR (*n* = 8) patients. Center line indicates the median, lower, and upper hinges represent the 25th and 75th percentiles, respectively, and whiskers denote 1.5× interquartile range. All differences with *P* < 0.10 are indicated. One-sided unpaired Wilcoxon test was used. **F** Scatter diagram showing a significantly positive correlation between expression level of CFP and apoptosis signature in TCGA-LUAD patients. *P* values were determined by two-sided Pearson correlation test. **G** Boxplots of the average expression of CFP in Mono_CX3CR1 cells in TN (*n* = 3), MPR (*n* = 4), and NMPR (*n* = 8) patients. One-sided *t*-test was used. **H** Violin and box plots of Mono_CX3CR1 signature in our validation cohort (9 patients were assessed as MPR and 12 as NMPR after surgery) before ICB + chemotherapy. One-sided unpaired Wilcoxon test was used. **I** Violin and box plots of Mono_CX3CR1 signature in responders (R) and non-responders (NR, removing SD patients) in advanced melanoma cohorts. Two-sided unpaired Wilcoxon test was used. **J** Kaplan–Meier survival curve of the signature of Mono_CX3CR1 in melanoma cohort 2. Survival curves were compared by the Log-Rank test. **K** Summary of selected ligand-receptor interactions from CellPhoneDB among Mono_CX3CR1 cells, cancer cells, and NK cells in MPR patients

The myeloid component in the TME exhibited remarkable heterogeneity and accordingly was categorized into 11 clusters, including 2 subtypes of monocyte, 6 subtypes of macrophage, and 3 subtypes of DC according to canonical marker genes (Fig. 5A and Additional file 2: Fig. S7A). We first focused on the monocytes. Mono_CX3CR1 highly expressed monocyte markers (FCN1, VCAN, S100A8, and S100A9), naïve marker (SELL), and lower MHC-II molecules (Fig. 5B, C), representing a “naïve-like” status. Mono_VEGFA had lower expression of monocyte markers, higher macrophage markers (MRC1, CD163, and MSR1), and MCH-II molecules than Mono_CX3CR1 cells, suggesting a “pre-macrophage” status (Fig. 5B, C). Trajectory analysis validated that Mono_VEGFA was an intermediate phenotype between naïve monocytes and macrophages (Fig. 5D). Mono_VEGFA highly expressed VEGFA, a pro-angiogenesis factor, reflecting an immunosuppressive phenotype (Fig. 5B, C). Consistently, Mono_VEGFA cells were decreased in MPR patients (Fig. 5E), and the expression of VEGFA was downregulated in MPR patients (Additional file 2: Fig. S7B).

Mono_CX3CR1 cells closely resembled non-classical “patrolling monocytes (PMos)” (CD14+CD16+CX3CR1+, Fig. 5C) [42]. Previous studies show that PMos scavenge tumor material from the lung vasculature and promote NK cell recruitment and activation [42]. Mono_CX3CR1 cells were of increased abundance in MPR patients (Fig. 5E). Mono_CX3CR1 cells also highly expressed CFP (Fig. 5C and Additional file 2: Fig. S7C), which has been reported to suppress breast cancer growth by inducing apoptosis [68]. The expression of CFP was strongly downregulated in TCGA-LUAD patients compared to normal lung tissues (Additional file 2: Fig. S7D), and higher CFP expression was associated with better survival (Additional file 2: Fig. S7E). The expression of CFP was significantly correlated with the apoptosis signature in TCGA-LUAD patients, and Mono_CX3CR1 cells in MPR patients had significantly higher expression of CFP than TN and NMPR patients (Fig. 5F, G).

We then investigate whether the gene signature from Mono_CX3CR1 monocytes (Additional file 3: Table S2) could be used as a biomarker to predict ICB response using bulk RNA-seq data. Higher Mono_CX3CR1 signature was observed in MPR patients in our validation cohort and responders from two independent melanoma cohorts (Fig. 5H, I). Higher Mono_CX3CR1 signature was associated with improved survival in Melanoma dataset 2 [34] (Fig. 5J). Cell-cell interaction analysis showed that the CX3CL1-CX3CR1 pair was significantly enriched between Mono_CX3CR1 cells and cancer cells from MPR patients (Fig. 5K), indicating that CX3CL1 expression in cancer cells attract Mono_CX3CR1 monocytes in MPR patients. Our analysis identified CX3CR1+ monocytes as associated with anti-tumor activity, and another immunosuppressive VEGFA+ monocytes as associated with poor response.

### Combined therapy expanded tissue-resident macrophages, reprogramed TAM into an M0 phenotype, and inhibited the immunosuppressive function in MPR patients

**Fig. 6:**
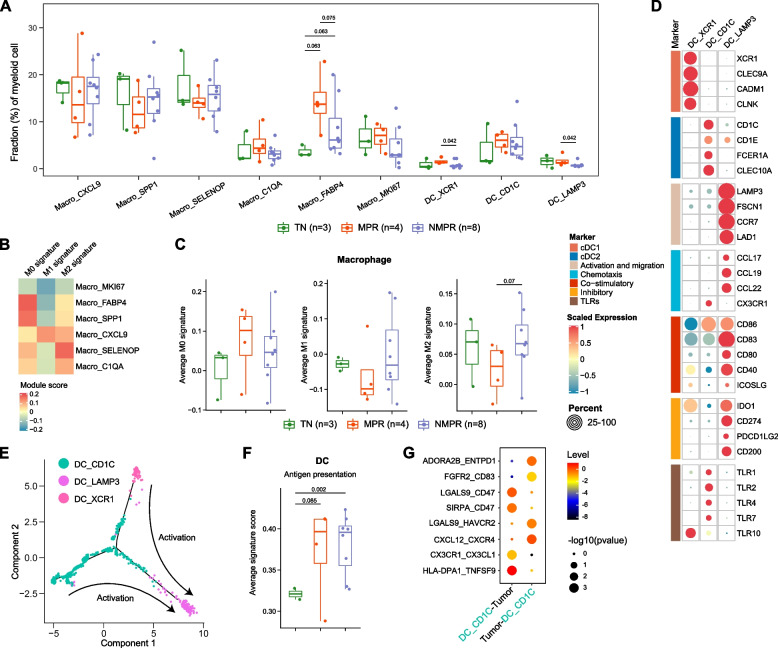
Macrophage and dendritic cell remodeling after therapy. **A** Boxplot showing cellular fractions of each macrophage and DC cluster in TN (*n* = 3), MPR (*n* = 4), and NMPR (*n* = 8) patients. Center line indicates the median, lower, and upper hinges represent the 25th and 75th percentiles, respectively, and whiskers denote 1.5× interquartile range. All differences with adjusted *P* < 0.10 are indicated. One-sided unpaired Wilcoxon test was used, and the *P* values were adjusted by the FDR method. **B** Heatmap of module scores of M0, M1, and M2 signatures among macrophage clusters. **C** Boxplots of average M0, M1, and M2 signature scores for macrophages in TN (*n* = 3), MPR (*n* = 4), and NMPR (*n* = 8) patients. One-sided *t*-test was used. **D** Heatmap of normalized expression of DC marker genes among clusters. **E** The developmental trajectory of DCs inferred by Monocle2. mregDCs (DC_LAMP3) could derive from cDC1s (DC_XCR1) or cDC2s (DC_CD1C). **F** Boxplots of average antigen presentation signature scores for DCs in TN (*n* = 2, one sample with less than 10 DC cells was removed), MPR (*n* = 4), and NMPR (*n* = 8) patients. One-sided *t*-test was used. **G** Summary of selected ligand-receptor interactions from CellPhoneDB among cancer cells and cDC2s (DC_CD1C) cells

We next focused on the macrophages. Macro_FABP4 cells are tissue-resident alveolar macrophages (AM) with the expression of the canonical AM markers (FABP4, MCEMP1 and MARCO; Fig. 5B) [69]. Concordant with its tissue repair function [70], Macro_FABP4 AMs were significantly elevated in post-treatment patients, although to a greater extent in MPR patients (Fig. 6A). Given tissue repair function of AT2 cells and the increase of AT2 cells after therapy (Additional file 2: Fig. S3F), AMs may work together with AT2 cells to regenerate normal lung structure.

Other macrophage subtypes show similarity to known tumor-associated macrophages (TAM). Macro_SPP1 macrophage has been previously reported to be associated with tumor angiogenesis [71] and to facilitate immune escape by upregulating PD-L1 [72]. Macro_SELENOP macrophage was previously reported to have anti-inflammation roles [73]. In contrast, Macro_CXCL9 cells overexpressed proinflammatory factors (CXCL9 and CXCL10) that could attract T cells, NK cells, and DCs [74]. The M2-like Macro_SPP1 macrophages decreased after therapy, while Macro_SELENOP macrophages had an increased trend in NMPR patients (Fig. 6A).

Macrophages are traditionally classified into three subtypes: M0 (non-polarized or neutral), M1 (proinflammatory or anti-tumor), or M2 (anti-inflammatory or pro-tumor). To better characterize the phenotype of macrophage subsets, we calculated M0, M1, and M2 signature scores based on the gene signatures from CIBERSORT [22]. As expected, the AM exhibited a M0-like phenotype, and both Macro_SPP1, Macro_SELENOP, and Macro_C1QA had a stronger M2 signature (Fig. 6B). Only Macro_CXCL9 cells showed a high M1 signature (Fig. 6B). Lineage tracing analysis suggested two distinct differentiation paths for monocytes. One path leads to M1-like Macro_CXCL9 cells and another path differentiated into M2-like Macro_SELENOP and Macro_SPP1 cells (Fig. 5D).

The M1 signature of macrophages would generally be expected to increase in responders after immunotherapy, while the M2 would generally be expected to decrease in responders after immunotherapy. Indeed, the M2 signature of macrophages decreased in MPR patients. However, both the M1 signature and M1-like subset (Macro_CXCL9) did not exhibit an increasing trend in MPR patients, and even decreased after therapy (Fig. 6A, C). Surprisingly, compared to the TN patients, the M0 signature increased in MPR patients (Fig. 6C). These analyses suggest that combined therapy induces expansion of tissue regenerative macrophages and reprograms TAM into a neutral instead of an anti-tumor phenotype in MPR patients, but inhibits the immunosuppressive function only in MPR patients. Suppressing the M2-like function of TAMs may be more effective than promoting M1-like activity to enhance immunotherapy response.

### Dendritic cells were activated by therapy and expanded in MPR patients

The DCs were classified into 3 subtypes, including conventional type I DCs (cDC1, DC_XCR1), conventional type II DCs (cDC2, DC_CD1C), and recently described LAMP3+ DCs (DC_LAMP3) (Fig. 6D) [75]. cDC1 and cDC2 have been previously reported to activate CD8+ T cells and CD4+ T cells, respectively [75]. LAMP3+ DC was reported to be “mature DCs enriched in immunoregulatory molecules” (mregDC) [76] due to expression of mature (LAMP3), migration (CCR7 and FSCN1) and immunoregulatory (CD274, PDCD1LG2, and CD200) markers, and downregulation of Toller-like receptors (TLRs).

mregDCs have been reported to interact with tumor-infiltrating Treg cells or to inhibit CD8+ T cell-mediated tumor immunity by IL-4 stimulation [76]. Trajectory analysis indicated that mregDCs may be generated from both cDC1s and cDC2s (Fig. 6E), consistent with recent reports that mregDCs are derived from cDC1s and cDC2s upon uptake of tumor antigens [30, 76]. After combined therapy, the fractions of cDC1s and cDC2s were increased in MPR patients (Fig. 6A). In addition, the antigen-presenting signature [23] of DCs was significantly increased after therapy, especially for MPR patients (Fig. 6F), suggesting that DCs were activated after therapy. The DC_CD1C cells expressed CX3CR1 (Fig. 6D), and the CX3CL1-CX3CR1 interaction was significantly enriched between DC_CD1C cells and cancer cells from MPR patients (Fig. 6G). The data indicates that the DCs are activated after therapy and recruited in MPR patients, which could contribute to the activation of CD8+ and CD4+ T cells in the TME.

### Aged neutrophils decreased in MPR patients and recruited SPP1+ TAMs through CCL3 and CCL4

**Fig. 7:**
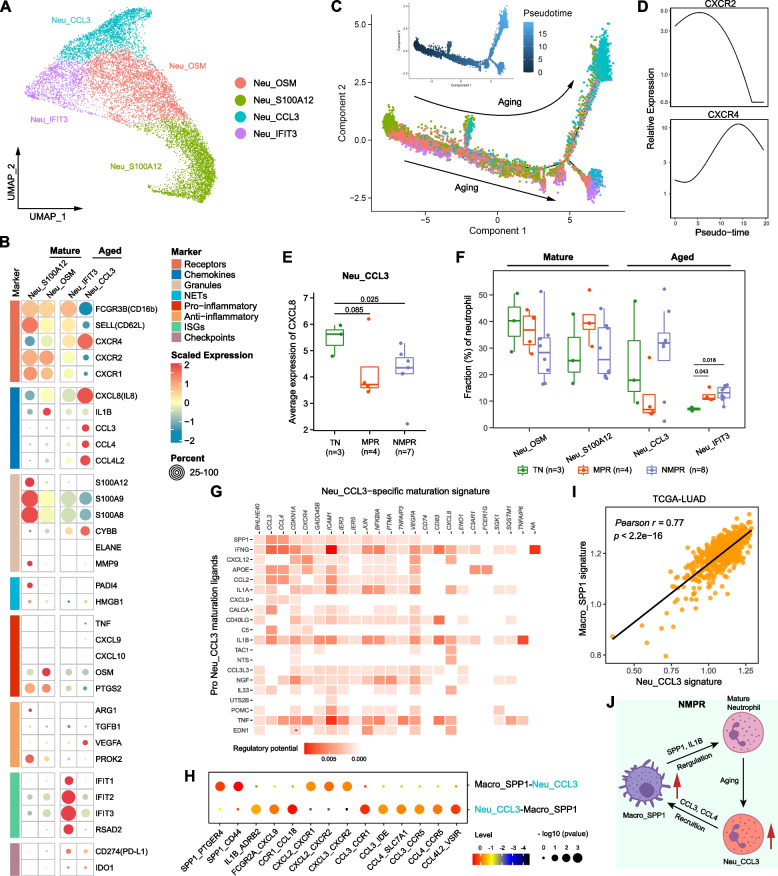
Neutrophil remodeling after therapy. **A** UMAP plot of neutrophils colored by clusters. **B** Heatmap of normalized expression of neutrophil marker genes among clusters. NETs, neutrophil extracellular traps; ISGs, interferon-stimulated genes. **C** The developmental trajectory of neutrophils inferred by Monocle2. The Neu_S100A12 cells were the roots of trajectory, and differentiated into Neu_CCL3 or Neu_IFIT3 cells. **D** Two-dimensional plots showing the dynamic expression of CXCR2 and CXCR4 during the neutrophils aging along the pseudo-time. **E** Boxplot of average expression of CXCL8 (IL8) in Neu_CCL3 cells in TN (*n* = 3), MPR (*n* = 4), and NMPR (*n* = 7, one sample with less than 10 Neu_CCL3 cells was removed) patients. One-sided *t*-test was used. **F** Boxplot showing cellular fractions of each neutrophil cluster in TN (*n* = 3), MPR (*n* = 4), and NMPR (*n* = 8) patients. Center line indicates the median, lower, and upper hinges represent the 25th and 75th percentiles, respectively, and whiskers denote 1.5× interquartile range. All differences with adjusted *P* < 0.05 are indicated. One-sided unpaired Wilcoxon test was used, and the *P* values were adjusted by the FDR method. **G** Heatmap showing potential ligands driving the phenotype of Neu_CCL3 neutrophils. **H** Summary of selected ligand-receptor interactions from CellPhoneDB between Neu_CCL3 neutrophils and Macro_SPP1 macrophages. **I** Scatter diagram showing a significantly positive correlation between the Neu_CCL3 signature and Macro_SPP1 signature in TCGA-LUAD patients. *P* values were determined by two-sided Pearson correlation test. **J** Inferred regulation network between Neu_CCL3 neutrophils and Macro_SPP1 macrophages

Neutrophils were divided into 4 subclusters, including 2 mature subsets (CD16+CXCR2^high^CXCR4^low^; Neu_S100A12 and Neu_OSM), and 2 aged subsets (CD16+CXCR2^low^CXCR4^high^; Neu_CCL3 and Neu_IFIT3) (Fig. 7A, B) [77]. Trajectory analysis indicated that the Neu_S100A12 subcluster was the root of the trajectory and that the Neu_CCL3 and Neu_IFIT3 subclusters were end-point states (Fig. 7C). Along this trajectory, the expression of CXCR2 decreased while CXCR4 increased in pseudo-time (Fig. 7D). Mature Neu_S100A12 neutrophils highly expressed genes associated with granules (S100A8, S100A9, and S100A12), which when released play a critical role in the proinflammatory response [78]. Mature Neu_OSM neutrophils were characterized by high expression of the cytokine OSM, which could promote production of proinflammatory molecules [79]. Among the aged subsets, Neu_CCL3 cells overexpressed multiple chemokines, including CCL3, CCL4, and CXCL8. Serum CXCL8 is reported to be a strong predictor of poor outcome in immunotherapy [80], consistent with lower expression of CXCL8 in Neu_CCL3 cells in MPR than in TN and NMPR patients (Fig. 7E). Neu_IFIT3 cells expressed interferon-stimulated genes (IFIT1-3, RSAD2, and MX1). Immune checkpoints (CD274 and IDO1) were upregulated in the aged clusters (Fig. 7B), reflecting an immunomodulatory phenotype. We found that the expression of the ELANE (elastase) gene, which has been reported to have an anti-cancer function in human neutrophils [81], was negative in all neutrophils (Fig. 7B). Pathway analysis indicated mature neutrophils exhibited enrichment of pathways of neutrophil activation, degranulation, and chemotaxis, while pathways of interferon signaling, translational initiation, and response to interleukin-1 were enriched in aged neutrophils (Additional file 2: Fig. S8A). Regulatory network analysis by SCENIC [31] showed that regulators KLF6, SPI1, FOS, and CEBPD were downregulated during neutrophil aging (Additional file 2: Fig. S8B).

To explore the reprogramming of neutrophils after combined therapy, we compared abundance of neutrophil subtype fractions before and after treatment. Aged neutrophils decreased after treatment in MPR patients and increased in NMPR patients (Additional file 2: Fig. S8C). When the neutrophils were divided into mature and aged phases in pseudo-time, results were similar (Additional file 2: Fig. S8D-E). Of all aged neutrophil subclusters, Neu_CCL3 cells were most depleted in MPR patients (Fig. 7F), indicating a suppressive phenotype in the TME.

Finally, we explored the mechanisms by which subsets of neutrophils were reprogrammed during therapy. NicheNet analysis predicted that SPP1, IFNγ, and IL1B ligands drive the specific phenotype of aged Neu_CCL3 cells (Fig. 7G), and IFNα ligands drive the phenotype of aged Neu_IFIT3 cells (Additional file 2: Fig. S8F). SPP1 was a key marker of Macro_SPP1 macrophages, so we evaluated the cell-cell interactions between aged Neu_CCL3 neutrophils and Macro_SPP1 macrophages. The SPP1-CD44 and IL1B-ADRB2 pairs were significantly enriched in the two cell types (Fig. 7H). It has been reported that CD44 regulates neutrophil phagocytosis and IL-8 production [82], and activation of ADRB2 (β2-adrenoceptor) could cause release of proinflammatory S100A8/A9 in neutrophils [83]. In addition, CCL3 and CCL4 secreted by aged Neu_CCL3 cells were predicted to recruit Macro_SPP1 macrophages by CCL3-CCR1, CCL3-CCR5, and CCL4-CCR5 pairs (Fig. 7H). The Neu_CCL3 signature was significantly correlated with the Macro_SPP1 signature in TCGA-LUAD patients (Fig. 7I). These indicates that Macro_SPP1 macrophages are involved in production of aged Neu_CCL3 neutrophils and that aged Neu_CCL3 neutrophils in turn recruit Macro_SPP1 macrophages (Fig. 7J). Lack of the Neu_CCL3-Macro_SPP1 interaction may lead to the decrease of Macro_SPP1 macrophages and Neu_CCL3 neutrophils in MPR patients (Figs. 6A and 7F).

## Discussion

**Fig. 8:**
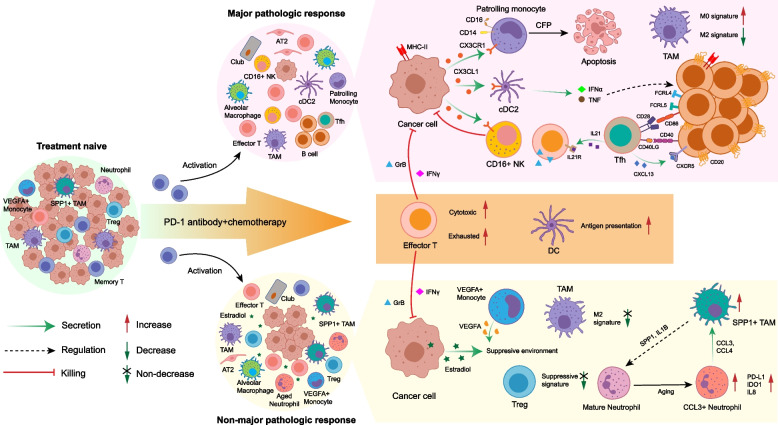
Summary of TME dynamics in NSCLC during ICB plus chemotherapy. After ICB plus chemotherapy, the phenotype of immune cells was remodeled, and normal epithelial cells expanded in the TME. The cytotoxic ability of effector T cells was significantly elevated; however, the exhausted markers were also increased. The memory CD8+ T cells were activated into an effector phenotype. Therapy enhanced the antigen-presenting function of DCs. Except these common features, major pathologic responders (MPRs), and non-MPRs had distinct characteristics of TME. The residual cancer cells in MPRs expressed MHC-II molecules to present tumor antigens themselves, and secreted CX3CL1 to recruit PMos, cDC2s, and CD16+ NK cells. PMos secreted CFP to promote apoptosis of cancer cells. Tfhs released CXCL13 to recruit CD20+ B cells and these B cells aggregated in the TME. IFNα and TNF from cDC2s drove the production of FCRL4+FCRL5+ memory B cells. The FCRL4+FCRL5+ memory B cells in turn activated Tfhs by CD86-CD28 and CD40-CD40LG interaction. Then, the activated Tfhs secreted IL21 to enhance release GrB from effector T cells though binding to IL21R. These interactions positively boosted the anti-tumor response. Meanwhile, suppressive Tregs and M2 signature of TAMs were decreased in MPRs. In non-MPRs, aberrant estrogen metabolism caused elevated estradiol in the TME. The TME in non-MPRs was still suppressive, with no decrease of M2 signature of TAMs and increase of VEGFA+ monocytes and suppressive signature of Tregs. In addition, the SPP1+ TAMs and CCL3+ neutrophils interacted with each other to promote expansion of themselves: SPP1+ TAMs secreted SPP1 and IL1B to induce the production of CCL3+ neutrophils, and CCL3+ neutrophils in turn to attract SPP1+ TAMs by CCL3 and CCL4

Improving response efficacy and identifying robust biomarkers are the major challenges for current immunotherapy. Although ICB therapy has been used in advanced NSCLC for years, many patients are refractory to treatment. The transcriptional characteristics underlying ICB resistance in NSCLC have not been characterized due to the difficulty of sample acquisition. The advent of neoadjuvant immunotherapy for resectable NSCLC provides the opportunity to collect tumor tissues before and after treatment and pathologic assessment of resected tumor tissues enables more precise response information compared to traditional RECIST classifications. Although two scRNA-seq studies regarding NSCLC immunotherapy have been reported recently, they focus on T cells [15, 56]. In this study, we examined single-cell transcriptomes from resectable NSCLC before and after combination treatment of PD-1 blockade and chemotherapy, and analyzed the entire TME across pathologic responses to investigate immune system and cancer responses to therapy (Fig. 8).

We uncovered transcriptional signatures of cancer cells specific to different pathologic responses. MHC-II genes were highly expressed in malignant cells from MPR patients. The important role of antigen presentation via the MHC-II pathway in MPR patients was also demonstrated by CD20+ B cells and cDC2s presenting tumor antigens to CD4+ T cells by MHC-II. Further, CD4+ Tfhs were activated by CD40-CD40LG and CD28-CD86 interactions in MPR patients. This suggests that although MHC-II expression is usually restricted to APCs, it could also be expressed intrinsically in a subset of cancer cells [84] or induced by IFNγ [43]. Given the low expression of MHC-II in TN patients, it is likely that the expression of MHC-II genes was induced by IFNγ secreted by effector T or NK cells as a result of therapy. Recent studies report that inhibition of histone deacetylases (HDAC) and mitogen-activated protein kinase kinase (MEK) enhanced MHC-II expression in NSCLC cell lines [84]. Therefore, promoting antigen presentation via the MHC-II pathway may be a strategy to enhance response to immunotherapy.

In NMPR patients, we observed overexpression of enzymes involved in estrogen metabolism in cancer cells, which resulted in the elevation of estradiol in serum. Previous studies have indirectly suggested a negative effect for estradiol in immunotherapy. A meta-analysis of 11,351 patients treated with ICB from 20 trials showed that ICB was significantly less effective in females than males and that females had no significant survival benefits in NSCLC [85]. Therefore, elevation of estradiol during therapy may be a biomarker for a poor response to therapy and raises the possibility that a regimen that combined anti-estrogen with ICB may improve response. Indeed, anti-estrogen had been explored in EGFR mutation positive NSCLC, although not in combination with ICB. Phase II trials failed to show a survival benefit for anti-estrogen combined with targeted therapy [86, 87], and therefore a phase III trial was not pursued. Future studies are needed to explore the efficacy of anti-estrogen combined with ICB.

Patients with different pathological responses showed remarkable differences in the TME remodeling after therapy. We hypothesize that there may be a “positive feedback” immune response in good responders (MPR patients), and a “negative feedback” response in poor responders (NMPR patients). ICB activates CD8+ T cells and NK cells to release multiple cytokines, thereby inducing expression of MHC-II in cancer cells and activating B cells and DCs to present tumor antigens. The reprogrammed cancer cells then present the tumor antigens to CD4+ Tfhs via MHC-II and release CX3CL1 to recruit NK cells, PMos and cDC2s. The “positive feedback” boosts the anti-tumor immune response. In good responders, simultaneous with the recruitment and activation of cytotoxic cells into the TME, immunosuppressive cells (Tregs, CCL3+ neutrophils, and SPP1+ TAMs) are reduced in the TME. In poor responders, anti-tumor immunity and cytotoxic cells are activated at the beginning of therapy, but the immunosuppressive cells were not adequately remodeled and become more abundant in the TME. Worse still, the CCL3+ neutrophils may interact with SPP1+ TAMs to promote expansion of each other. In poor responders, cancer cells limit the immune response by increasing estradiol levels. Adjunctive therapies that decrease the immunosuppressive cells or factors in the TME to allow a return to a positive feedback cycle may greatly improve response to immunotherapy.

There were three major limitations to this study. First, due to the difficulty of acquiring NSCLC tumor samples, the patient sample size was small. Therefore, it was difficult to achieve statistical separation to support of conclusions on the patient level, and there was a risk that patient variability may be more pronounced than biological effects. Second, the patients in this study received combination immunotherapy and chemotherapy. Therefore, mechanisms revealed by our study may provide novel targets or biomarkers that are independent of a specific therapy. Third, because neutrophils were nearly absent in public scRNA-seq data of lung cancer, independent validations of the hypothesis regarding CCL3+ neutrophils and SPP1+ TAMs were not available. Interestingly, a recent study of liver tumor also described that the co-enrichment of CCL3+ neutrophils and SPP1+ macrophages was associated with poor prognosis [88], indicating a similar mechanism across different cancer types.

## Conclusions

scRNA-seq analysis of resectable NSCLC revealed the dynamics of the TME before and after neoadjuvant ICB combined with chemotherapy and distinct TME properties between good responders and poor responders. We identified serum estradiol and two cell types in the TME (FCRL4+ FCRL5+ memory B cells and CD16+CX3CR1+ monocytes) that could serve as biomarkers for treatment response. Further, our study captured a high proportion of neutrophils, revealing great heterogeneity during immunotherapy. The dataset will be a valuable ongoing resource for cancer and neutrophil biology.

## Supplementary Information

**Additional file 1: Table S1.** Clinical information of tumor and blood samples used in the study.

**Additional file 2: Fig. S1.** Sample collection, single-cell clustering and marker gene visualization for major lineages. **Fig. S2.** Immunohistochemistry (IHC) staining of immune cells. **Fig. S3.** Cluster marker genes, tumor cell identification, cellular fractions and hematoxylin eosin (H&E) staining for epithelial cells. **Fig. S4.** Upregulated genes and signatures for malignant cells among TN, MPR and NMPR patients and serum estradiol dynamics. **Fig. S5.** Marker genes, differentially expressed genes and RNA velocity for T/NK subsets. **Fig. S6.** Marker genes, immunofluorescence, and Nichenet analysis for B-cell subsets. **Fig. S7.** Marker genes and survival analysis for myeloid subsets. **Fig. S8.** Monocle, SCENIC and cellular fraction analysis for neutrophil subsets.

**Additional file 3: Table S2.** Gene list of function modules.

**Additional file 4: Table S3.** Intensity matrix of steroids.

## Data Availability

Raw single-cell RNA-seq and bulk RNA-seq data are deposited to Genome Sequence Archive of the BIG Data Center at the Beijing Institute of Genomics, Chinese Academy of Science, under accession number HRA001033 (https://ngdc.cncb.ac.cn/gsa-human/browse/HRA001033) [89]. The count matrix of scRNA-seq and TPM matrix of bulk RNA-seq can be obtained from Gene Expression Omnibus, under accession number GSE207422 (https://www.ncbi.nlm.nih.gov/geo/query/acc.cgi?acc=GSE207422) [90]. No new algorithms were developed for this manuscript. Code used for all processing and analysis is available on GitHub (https://github.com/Junjie-Hu/NSCLC-immunotherapy) [91].
