# Supplementary material for: Tumor microenvironment remodeling after neoadjuvant immunotherapy in non-small cell lung cancer revealed by single-cell RNA sequencing
Source: Genome Med. 2023 Mar 3;15:14. doi: 10.1186/s13073-023-01164-9 (PMC9985263; doi:10.1186/s13073-023-01164-9)
Supplement: Supplementary file 2 — Additional file 2: Fig. S1. Sample collection, single-cell clustering and marker gene visualization for major lineages. Fig. S2. Immunohistochemistry (IHC) staining of immune cells. Fig. S3. Cluster marker genes, tumor cell identification, cellular fractions and hematoxylin eosin (H&E) staining for epithelial cells. Fig. S4. Upregulated genes and signatures for malignant cells among TN, MPR and NMPR patients and serum estradiol dynamics. Fig. S5. Marker genes, differentially expressed genes and RNA velocity for T/NK subsets. Fig. S6. Marker genes, immunofluorescence, and Nichenet analysis for B-cell subsets. Fig. S7. Marker genes and survival analysis for myeloid subsets. Fig. S8. Monocle, SCENIC and cellular fraction analysis for neutrophil subsets. [file 13073_2023_1164_MOESM2_ESM.pdf]

Supplementary Information

Additional file 2: Supplementary Materials for: Tumor Microenvironment Remodeling after Neoadjuvant Immunotherapy in Non-small Cell Lung Cancer Revealed by Single-Cell RNA Sequencing. This file includes Supplementary Figures: Fig. S1-S8

Fig. S1: Sample collection, single-cell clustering and marker gene visualization for major lineages.
Fig. S2: Immunohistochemistry (IHC) staining of immune cells.
Fig. S3: Cluster marker genes, tumor cell identification, cellular fractions and hematoxylin eosin (H&E) staining for epithelial cells.
Fig. S4: Upregulated genes and signatures for malignant cells among TN, MPR and NMPR patients and serum estradiol dynamics.
Fig. S5: Marker genes, differentially expressed genes and RNA velocity for T/NK subsets
Fig. S6: Marker genes, immunofluorescence, and Nichenet analysis for B-cell subsets.
Fig. S7: Marker genes and survival analysis for myeloid subsets.
Fig. S8: Monocle, SCENIC and cellular fraction analysis for neutrophil subsets.

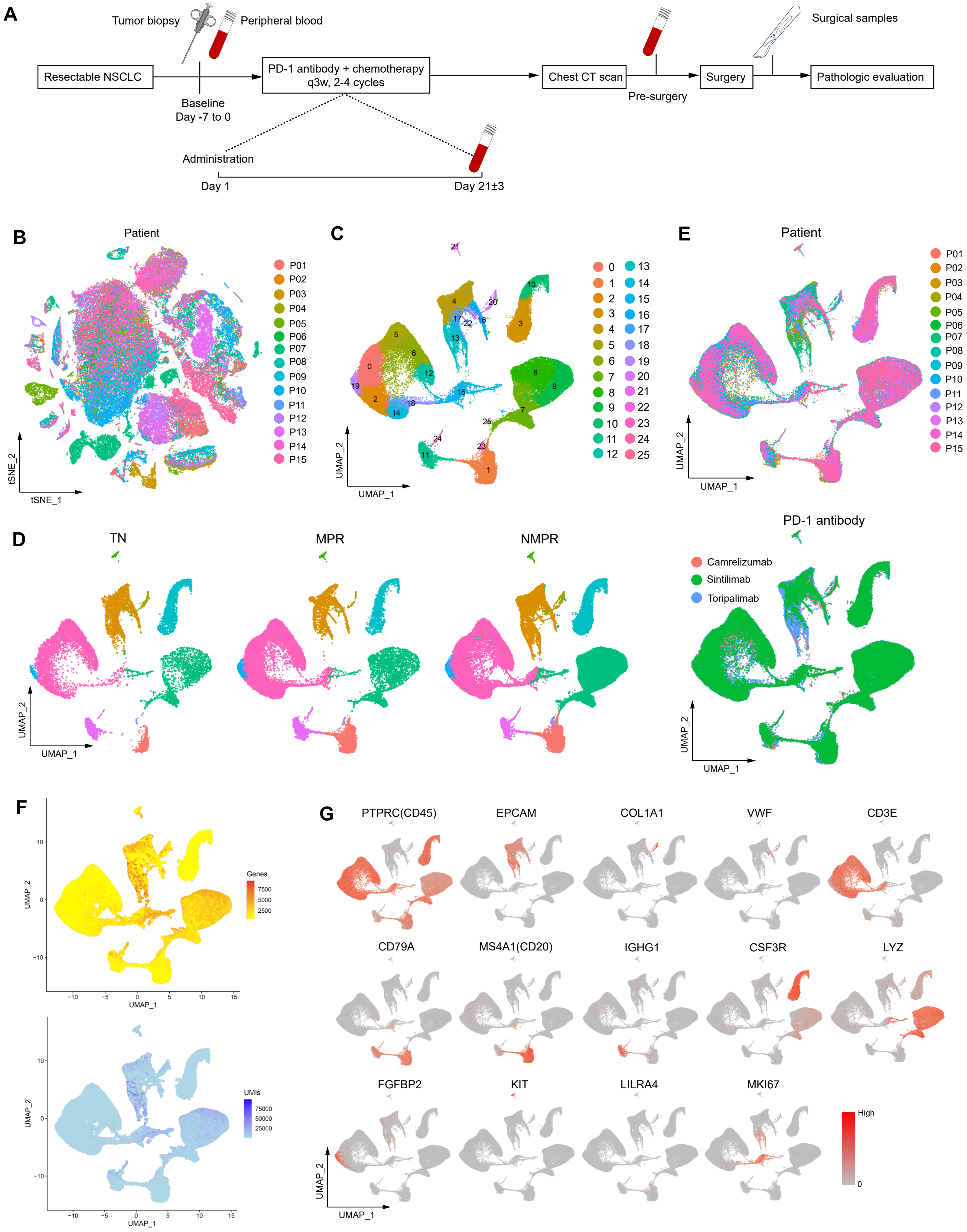
A
Tumor biopsy
Peripheral blood
Surgical samples
Resectable NSCLC
PD-1 antibody + chemotherapy
q3w, 2-4 cycles
Chest CT scan
Surgery
Pathologic evaluation
Baseline
Day -7 to 0
Pre-surgery
Administration
Day 1
Day 21±3
B
Patient
P01
P02
P03
P04
P05
P06
P07
P08
P09
P10
P11
P12
P13
P14
P15
tSNE_1
tSNE_2
C
UMAP_1
UMAP_2
E
Patient
D
TN
MPR
NMPR
PD-1 antibody
Camrelizumab
Sintilimab
Toripalimab
F
Genes
7500
5000
2500
UMIs
75000
50000
25000
G
PTPRC(CD45)
EPCAM
COL1A1
VWF
CD3E
CD79A
MS4A1(CD20)
IGHG1
CSF3R
LYZ
FGFBP2
KIT
LILRA4
MKI67
High
0

Fig. S1: Sample collection, single-cell clustering and marker gene visualization for major lineages.

A, Schematic representation of trial treatment.

B, tSNE plot showing batch effects from patients.

C, UMAP plot of all cells categorized into 26 clusters.

D, UMAP plots of the cells from treatment naïve (TN), major pathologic response (MPR) and non-major pathologic response (NMPR) patients.

E, UMAP plots of all cells colored by patient origin and PD-1 antibody.

F, UMAP plots showing the number of detected genes and UMIs for all cells.

G, UMAP plots showing the expression of selected marker genes for the defined cell types.

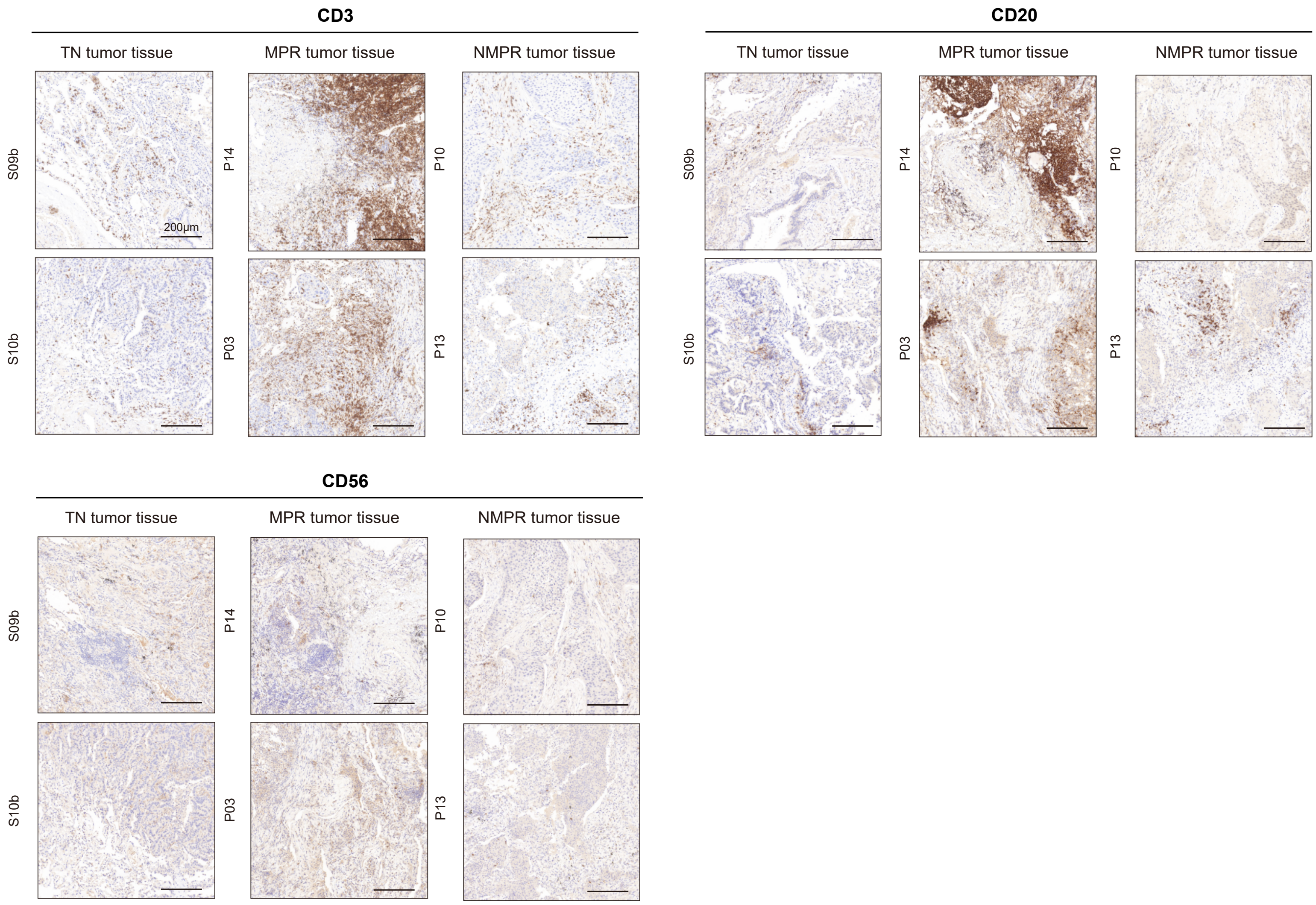

Fig. S2: Immunohistochemistry (IHC) staining of immune cells. Representative IHC staining of T (CD3), NK (CD56) and B (CD20) cells in another two TN, MPR and NMPR patients, respectively.

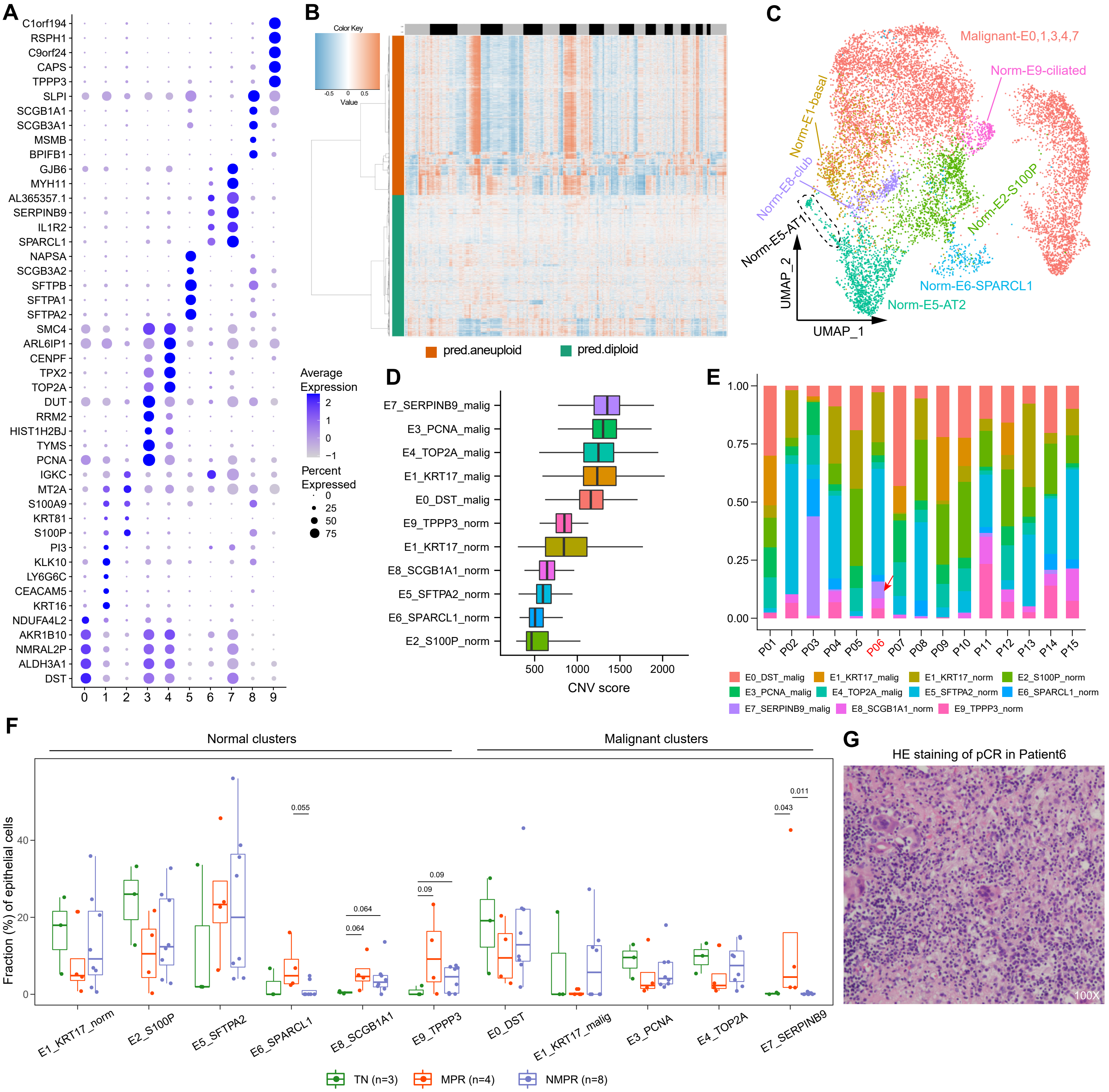

Fig. S3: Cluster marker genes, tumor cell identification, cellular fractions and hematoxylin eosin (H&E) staining f or epithelial cells. A, Dot plots of top 5 marker genes for each epithelial cluster. B, Heatmap showing large-scale CNVs for individual epithelia. Each row represented a cell and the columns represented chromosomal regions. The cells in orange were predicted to be malignant cells and green were normal cells. C, UMAP plots of epithelia colored by malignant (in red) and normal clusters. The cells in the black dash line were alveolar type I (AT1) cells. D, Box plots of CNV scores for each epithelial cluster. Center line indicates the median, lower and upper hinges represent the 25th and 75th percentiles, respectively, and whiskers denote 1.5 × interquartile range. Two-sided unpaired Wilcoxon test was used. E, The proportions of the cells in different clusters for each patient. The red arrow marked the malignant E7_SERPINB9 cells in Patients6 whose tumor was assessed to be no viable tumor cells after therapy (pCR). F, Boxplot showing cellular fractions of each epithelial cluster in TN (n=3), MPR (n=4) and NMPR (n=8) patients. All differences with adjusted $P < 0.05$ are indicated; One-sided unpaired Wilcoxon test was used, and the P values were adjusted by the false discovery rate (FDR) method. G, H&E staining image of P06 whose tumor was assessed to be pCR.

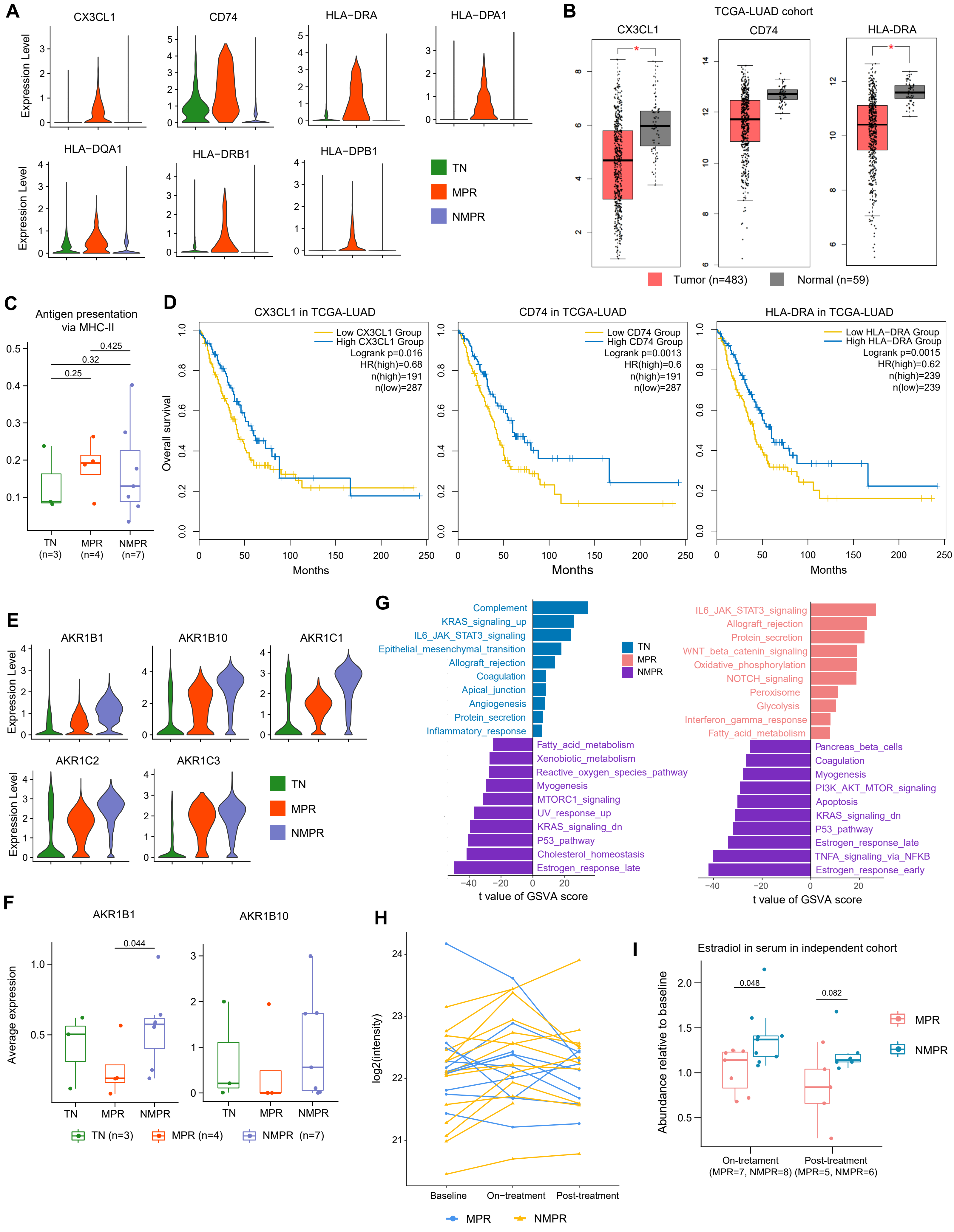
A
CX3CL1
CD74
HLA−DRA
HLA−DPA1
HLA−DQA1
HLA−DRB1
HLA−DPB1
Expression Level
TN
MPR
NMPR
B
TCGA-LUAD cohort
CX3CL1
CD74
HLA-DRA
Tumor (n=483)
Normal (n=59)
C
Antigen presentation via MHC-II
0.425
0.32
0.25
TN (n=3)
MPR (n=4)
NMPR (n=7)
D
CX3CL1 in TCGA-LUAD
Low CX3CL1 Group
High CX3CL1 Group
Logrank p=0.016
HR(high)=0.68
n(high)=191
n(low)=287
CD74 in TCGA-LUAD
Low CD74 Group
High CD74 Group
Logrank p=0.0013
HR(high)=0.6
n(high)=191
n(low)=287
HLA-DRA in TCGA-LUAD
Low HLA−DRA Group
High HLA−DRA Group
Logrank p=0.0015
HR(high)=0.62
n(high)=239
n(low)=239
Overall survival
Months
E
AKR1B1
AKR1B10
AKR1C1
AKR1C2
AKR1C3
Expression Level
TN
MPR
NMPR
F
AKR1B1
AKR1B10
0.044
Average expression
TN
MPR
NMPR
TN (n=3)
MPR (n=4)
NMPR (n=7)
G
Complement
KRAS_signaling_up
IL6_JAK_STAT3_signaling
Epithelial_mesenchymal_transition
Allograft_rejection
Coagulation
Apical_junction
Angiogenesis
Protein_secretion
Inflammatory_response
Fatty_acid_metabolism
Xenobiotic_metabolism
Reactive_oxygen_species_pathway
Myogenesis
MTORC1_signaling
UV_response_up
KRAS_signaling_dn
P53_pathway
Cholesterol_homeostasis
Estrogen_response_late
TN
MPR
NMPR
t value of GSVA score
IL6_JAK_STAT3_signaling
Allograft_rejection
Protein_secretion
WNT_beta_catenin_signaling
Oxidative_phosphorylation
NOTCH_signaling
Peroxisome
Glycolysis
Interferon_gamma_response
Fatty_acid_metabolism
Pancreas_beta_cells
Coagulation
Myogenesis
PI3K_AKT_MTOR_signaling
Apoptosis
KRAS_signaling_dn
P53_pathway
Estrogen_response_late
TNFA_signaling_via_NFKB
Estrogen_response_early
t value of GSVA score
H
log2(intensity)
Baseline
On−treatment
Post-treatment
MPR
NMPR
I
Estradiol in serum in independent cohort
0.048
0.082
Abundance relative to baseline
On-tretament (MPR=7, NMPR=8)
Post-treatment (MPR=5, NMPR=6)
MPR
NMPR

Fig. S4: Upregulated genes and signatures for malignant cells among TN, MPR and NMPR patients and serum estradiol dynamics.

A, Violin plots of upregulated genes in malignant cells in MPR patients.

B, Boxplots of the expression of CX3CL1, CD74 and HLA-DRA in TCGA-LUAD and normal lung tissues. Center line indicates the median, lower and upper hinges represent the 25th and 75th percentiles, respectively, and whiskers denote 1.5 × interquartile range. Two-sided unpaired Wilcoxon test was used. *$P < 0.05$, ANOVA test.

C, Boxplot of the average signature scores of antigen presentation via MHC-II for cancer cells in TN (n=3), MPR (n=4) and NMPR (n=7) patients. One-sided t-test was used.

D, Kaplan–Meier survival curve of the expression of CX3CL1, CD74 and HLA-DRA in TCGA lung adenocarcinoma (LUAD) cohort. Two-sided log-rank test was used.

E,Violin plots of upregulated genes in malignant cells in NMPR patients.

F, Boxplots of the average expression of ARK1B1 and ARK1B10 in malignant cells in TN (n=3), MPR (n=4) and NMPR (n=7) patients. One-sided t-test was used.

G, Top 10 differences in pathway activities scored per cell by GSVA comparing malignant cells between TN (n=1077) and NMPR (n=5368) patients (left panel) and between MPR (n=997) and NMPR patients (right panel).

H, Line plot of estradiol abundance (log2 intensity) in baseline, on-treatment and post-treatment for each patient.

I, Boxplots of the β-estradiol abundance relative to baseline in 15 patients independent against scRNA-seq cohort (7 patients were assessed as MPR and 8 as NMPR after surgery) at on-treatment and post-treatment timepoint.

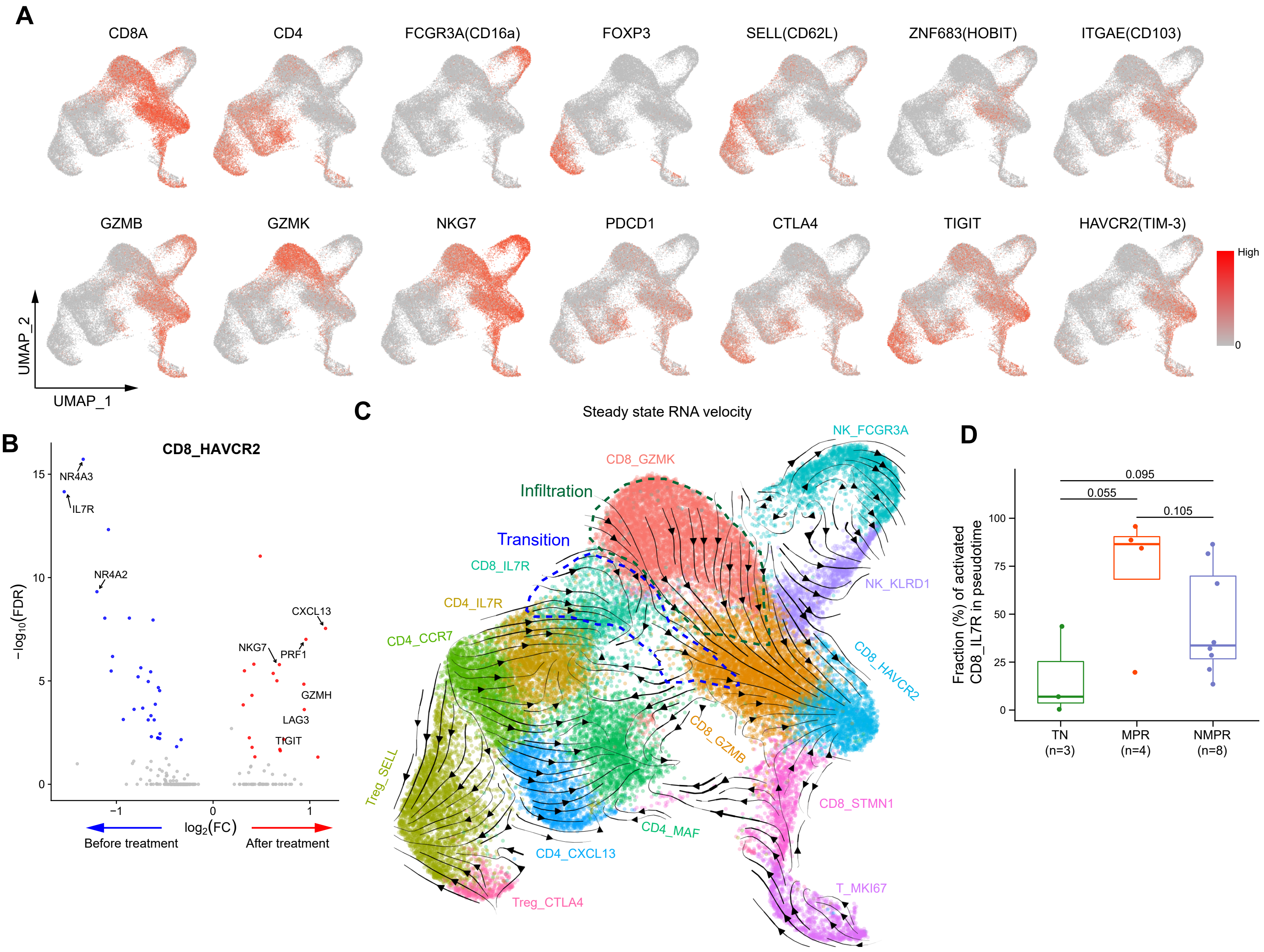

Fig. S5: Marker genes, differentially expressed genes and RNA velocity for T/NK subsets

A, UMAP plots showing the expression of selected canonical marker genes for T/NK cells.

B, Volcano plot of differentially expressed genes of CD8_HAVCR2 cells before and after therapy.

C, Steady-state RNA velocity of T/NK cell clusters. The blue dash line marked the memory T cells transition into cytotoxic cells, and the blackish-green dash line marked the differentiation of effector memory cells into residual memory cells and exhausted cells.

D, Box plots of fractions of activated memory T (CD8_IL7R) cells in pseudo-time in TN (n=3), MPR (n=4) and NMPR (n=8) patients. Center line indicates the median, lower and upper hinges represent the 25th and 75th percentiles, respectively, and whiskers denote 1.5× interquartile range. One-sided unpaired Wilcoxon test was used.

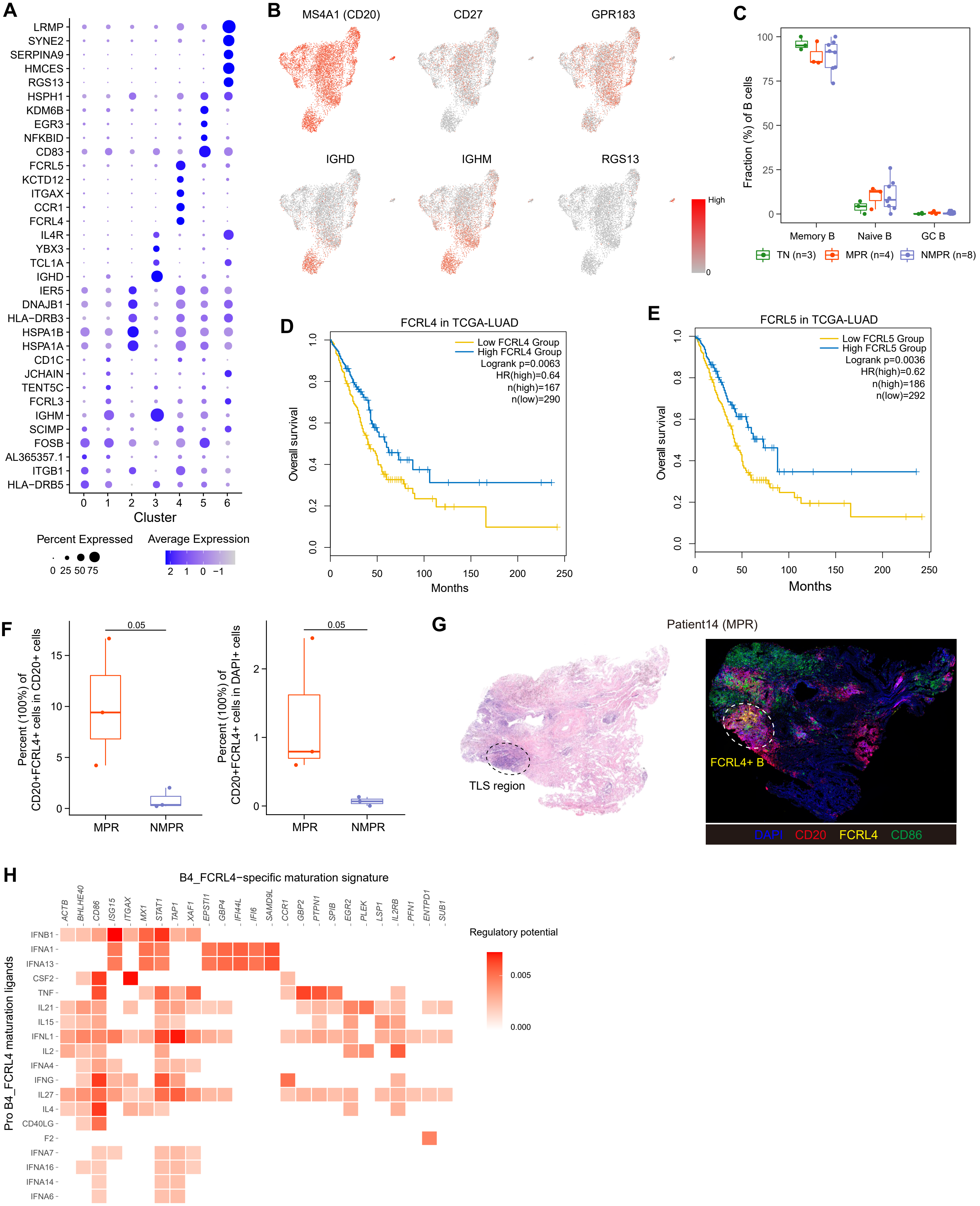
A
LRMP
SYNE2
SERPINA9
HMCES
RGS13
HSPH1
KDM6B
EGR3
NFKBID
CD83
FCRL5
KCTD12
ITGAX
CCR1
FCRL4
IL4R
YBX3
TCL1A
IGHD
IER5
DNAJB1
HLA−DRB3
HSPA1B
HSPA1A
CD1C
JCHAIN
TENT5C
FCRL3
IGHM
SCIMP
FOSB
AL365357.1
ITGB1
HLA−DRB5
0 1 2 3 4 5 6
Cluster
Percent Expressed
0 25 50 75
Average Expression
2 1 0 −1
B
MS4A1 (CD20)
CD27
GPR183
IGHD
IGHM
RGS13
High
0
C
Fraction (%) of B cells
100
75
50
25
0
Memory B
Naive B
GC B
TN (n=3)
MPR (n=4)
NMPR (n=8)
D
FCRL4 in TCGA-LUAD
Low FCRL4 Group
High FCRL4 Group
Logrank p=0.0063
HR(high)=0.64
n(high)=167
n(low)=290
Overall survival
Months
E
FCRL5 in TCGA-LUAD
Low FCRL5 Group
High FCRL5 Group
Logrank p=0.0036
HR(high)=0.62
n(high)=186
n(low)=292
Overall survival
Months
F
0.05
Percent (100%) of CD20+FCRL4+ cells in CD20+ cells
MPR
NMPR
0.05
Percent (100%) of CD20+FCRL4+ cells in DAPI+ cells
MPR
NMPR
G
Patient14 (MPR)
TLS region
FCRL4+ B
DAPI CD20 FCRL4 CD86
H
B4_FCRL4−specific maturation signature
ACTB
BHLHE40
CD86
ISG15
ITGAX
MX1
STAT1
TAP1
XAF1
EPSTI1
GBP4
IFI44L
IFI6
SAMD9L
CCR1
GBP2
PTPN1
SPIB
EGR2
PLEK
LSP1
IL2RB
PFN1
ENTPD1
SUB1
Pro B4_FCRL4 maturation ligands
IFNB1
IFNA1
IFNA13
CSF2
TNF
IL21
IL15
IFNL1
IL2
IFNA4
IFNG
IL27
IL4
CD40LG
F2
IFNA7
IFNA16
IFNA14
IFNA6
Regulatory potential
0.005
0.000

Fig. S6: Marker genes, immunofluorescence, and Nichenet analysis for B-cell subsets.

A, Dot plot of top 5 marker genes for each B cluster.

B, UMAP plots showing the expression of selected marker genes for the defined cell types.

C, Boxplot showing cellular fractions of each B cluster in TN (n=3), MPR (n=4) and NMPR (n=8) patients. Center line indicates the median value, lower and upper hinges represent the 25th and 75th percentiles, respectively, and whiskers denote 1.5× interquartile range. All P values > 0.05, one-sided unpaired Wilcoxon test.

D, Kaplan–Meier survival curve of the expression of FCRL4 in TCGA-LUAD cohort.

E, Kaplan–Meier survival curve of the expression of FCRL5 in TCGA-LUAD cohort.

F, Quantification of FCRL4+ B cells from the multiplex immunofluorescence images.

G, HE and multiplex IF showing the location of tertiary lymphoid structures (TLS) and FCRL4+ B cells.

H, Heatmap showing potential ligands driving the phenotype of B4_FCRL4 cells.

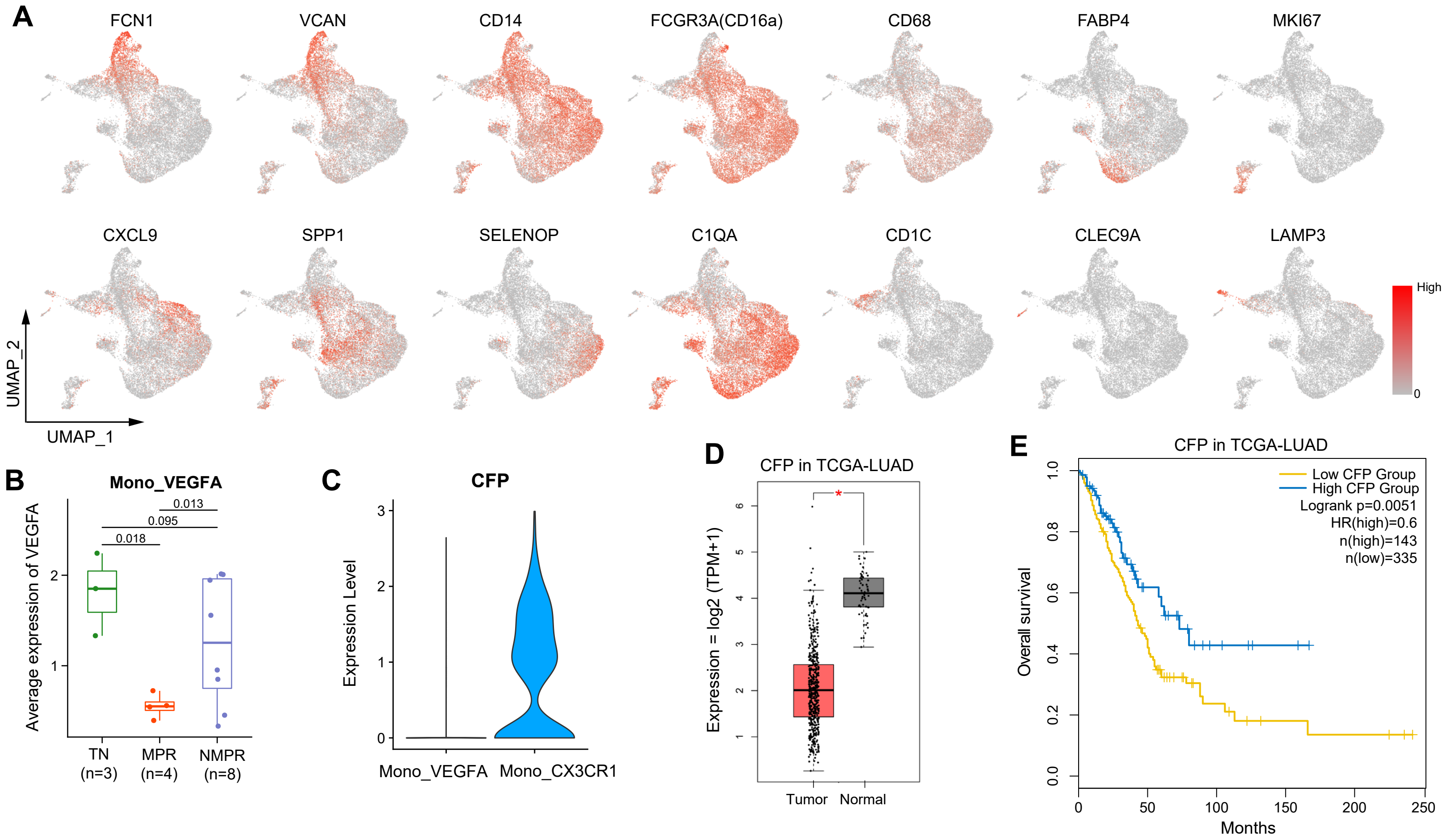

Fig. S7: Marker genes and survival analysis for myeloid subsets.

A, UMAP plots showing the expression of selected canonical marker genes for myeloid cells.

B, Boxplots of the average expression of VEGFA in Mono_ VEGFA cells in TN (n=3), MPR (n=4) and NMPR (n=8) patients. Center line indicates the median, lower and upper hinges represent the 25th and 75th percentiles, respectively, and whiskers denote 1.5 × interquartile range. Two-sided unpaired Wilcoxon test was used. One-sided t-test was used.

C, Violin plot of the expression of CFP in Mono_VEGFA and Mono_CX3CR1 cells.

D, Boxplots of the expression of CFP in TCGA-LUAD and normal lung tissues. *P < 0.05, ANOVA test.

E, Kaplan–Meier survival curve of the expression of CFP in TCGA-LUAD cohort.

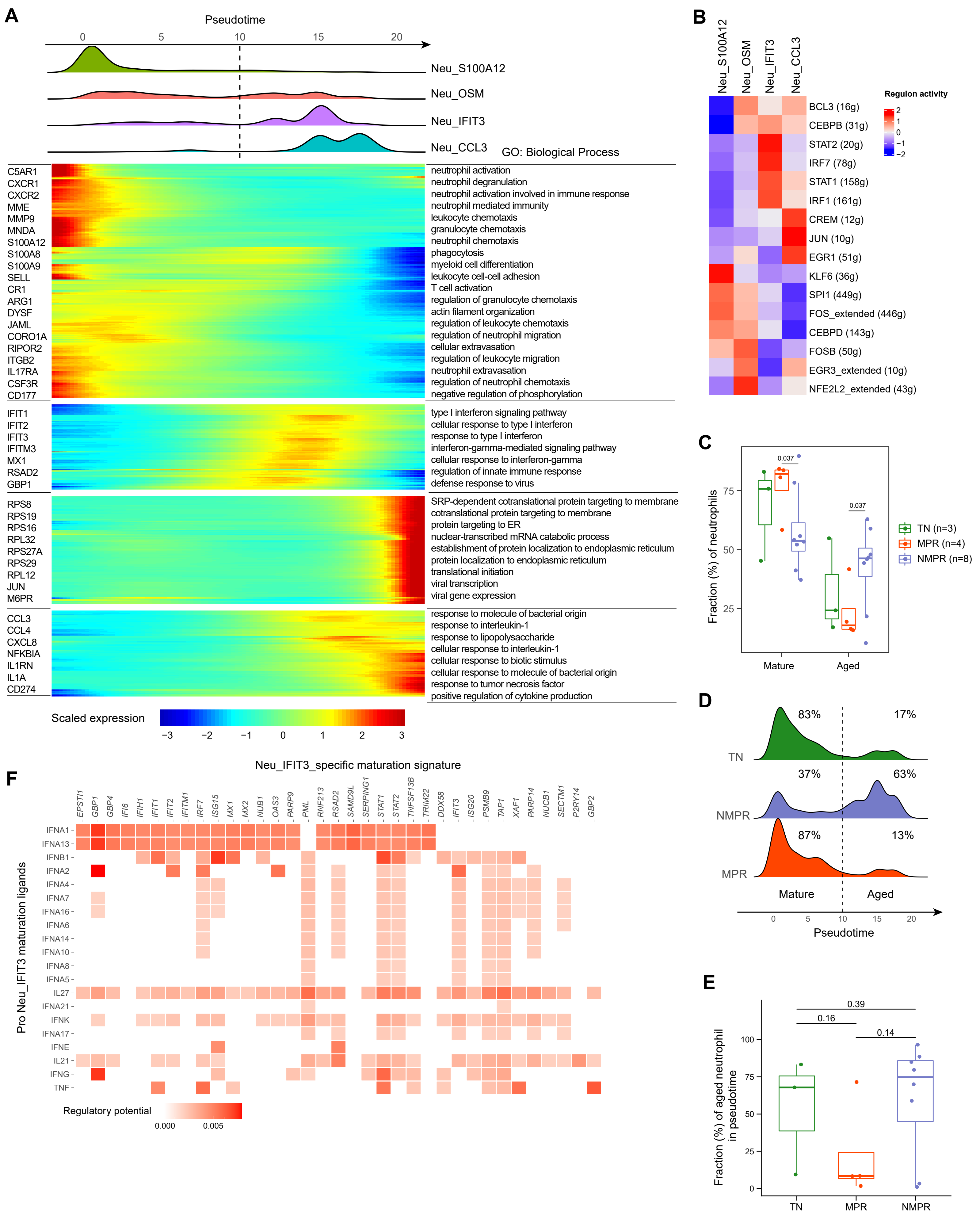
A
Pseudotime
0 5 10 15 20
Neu_S100A12
Neu_OSM
Neu_IFIT3
Neu_CCL3
GO: Biological Process
C5AR1
CXCR1
CXCR2
MME
MMP9
MNDA
S100A12
S100A8
S100A9
SELL
CR1
ARG1
DYSF
JAML
CORO1A
RIPOR2
ITGB2
IL17RA
CSF3R
CD177
neutrophil activation
neutrophil degranulation
neutrophil activation involved in immune response
neutrophil mediated immunity
leukocyte chemotaxis
granulocyte chemotaxis
neutrophil chemotaxis
phagocytosis
myeloid cell differentiation
leukocyte cell-cell adhesion
T cell activation
regulation of granulocyte chemotaxis
actin filament organization
regulation of leukocyte chemotaxis
regulation of neutrophil migration
cellular extravasation
regulation of leukocyte migration
neutrophil extravasation
regulation of neutrophil chemotaxis
negative regulation of phosphorylation
IFIT1
IFIT2
IFIT3
IFITM3
MX1
RSAD2
GBP1
type I interferon signaling pathway
cellular response to type I interferon
response to type I interferon
interferon-gamma-mediated signaling pathway
cellular response to interferon-gamma
regulation of innate immune response
defense response to virus
RPS8
RPS19
RPS16
RPL32
RPS27A
RPS29
RPL12
JUN
M6PR
SRP-dependent cotranslational protein targeting to membrane
cotranslational protein targeting to membrane
protein targeting to ER
nuclear-transcribed mRNA catabolic process
establishment of protein localization to endoplasmic reticulum
protein localization to endoplasmic reticulum
translational initiation
viral transcription
viral gene expression
CCL3
CCL4
CXCL8
NFKBIA
IL1RN
IL1A
CD274
response to molecule of bacterial origin
response to interleukin-1
response to lipopolysaccharide
cellular response to interleukin-1
cellular response to biotic stimulus
cellular response to molecule of bacterial origin
response to tumor necrosis factor
positive regulation of cytokine production
Scaled expression
−3 −2 −1 0 1 2 3
B
Neu_S100A12
Neu_OSM
Neu_IFIT3
Neu_CCL3
Regulon activity
2 1 0 −1 −2
BCL3 (16g)
CEBPB (31g)
STAT2 (20g)
IRF7 (78g)
STAT1 (158g)
IRF1 (161g)
CREM (12g)
JUN (10g)
EGR1 (51g)
KLF6 (36g)
SPI1 (449g)
FOS_extended (446g)
CEBPD (143g)
FOSB (50g)
EGR3_extended (10g)
NFE2L2_extended (43g)
C
Fraction (%) of neutrophils
75
50
25
0.037
0.037
Mature
Aged
TN (n=3)
MPR (n=4)
NMPR (n=8)
D
TN
83%
17%
NMPR
37%
63%
MPR
87%
13%
Mature
Aged
0 5 10 15 20
Pseudotime
E
Fraction (%) of aged neutrophil in pseudotime
100
75
50
25
0
0.39
0.16
0.14
TN
MPR
NMPR
F
Neu_IFIT3_specific maturation signature
EPSTI1 GBP1 GBP4 IFI6 IFIH1 IFIT1 IFIT2 IFITM1 IRF7 ISG15 MX1 MX2 NUB1 OAS3 PARP9 PML RNF213 RSAD2 SAMD9L SERPING1 STAT1 STAT2 TNFSF13B TRIM22 DDX58 IFIT3 ISG20 PSMB9 TAP1 XAF1 PARP14 NUCB1 SECTM1 P2RY14 GBP2
Pro Neu_IFIT3 maturation ligands
IFNA1
IFNA13
IFNB1
IFNA2
IFNA4
IFNA7
IFNA16
IFNA6
IFNA14
IFNA10
IFNA8
IFNA5
IL27
IFNA21
IFNK
IFNA17
IFNE
IL21
IFNG
TNF
Regulatory potential
0.000 0.005

Fig. S8: Monocle, SCENIC and cellular fraction analysis for neutrophil subsets.

A, Heatmap of the top differential genes in neutrophils along the pseudo-time (lower panel). The distribution of neutrophils during the transition along with the pseudo-time (upper panel). Subsets were labeled by colors (upper panel).

B, Heatmap showing transcription factor activity for each neutrophil subset.

C, Boxplot showing cellular fractions of mature (Neu_S100A12 and Neu_OSM) and aged (Neu_CCL3 and Neu_IFIT3) cells in TN (n=3), MPR (n=4) and NMPR (n=8) patients. Center line indicates the median value, lower and upper hinges represent the 25th and 75th percentiles, respectively, and whiskers denote 1.5× interquartile range. All differences with $P < 0.05$ are indicated; One-sided unpaired Wilcoxon test was used.

D, The distribution of neutrophils during the transition (divided into 2 phases: mature and aged) along with the pseudo-time in TN, MPR and NMPR patients.

E, Box plots of fractions of aged neutrophils in pseudo-time in TN (n=3), MPR (n=4) and NMPR (n=8) patients. One-sided unpaired Wilcoxon test was used. F, Heatmap showing potential ligands driving the phenotype of Neu_IFIT3 cells.
